# Prevalence and Risk Factors of Renal Artery Stenosis in Patients Undergoing Simultaneous Coronary and Renal Artery Angiography: A Systematic Review and Meta-Analysis of 31,689 Patients from 31 Studies

**DOI:** 10.3390/diseases12090208

**Published:** 2024-09-11

**Authors:** Konstantin Schwarz, Ida Straume Bah, Maximilian Will, Chun Shing Kwok, Julia Mascherbauer, Marko Kumric, Josko Bozic, Josip A. Borovac

**Affiliations:** 1 Department of Internal Medicine 3, University Hospital St. Pölten, Karl Landsteiner University of Health Sciences, 3500 Krems, Austria; konstantin.schwarz@gmx.net (K.S.); julia.mascherbauer@meduniwien.ac.at (J.M.); 2 Department of Pathophysiology, University of Split School of Medicine (USSM), 21000 Split, Croatia; ida.s.bah@gmail.com (I.S.B.); marko.kumric@mefst.hr (M.K.); josko.bozic@mefst.hr (J.B.); 3 Karl Landsteiner Institute for Cardiometabolics, Karl Landsteiner Society, 3500 St. Poelten, Austria; 4 Department of Cardiology, Leighton Hospital, Mid Cheshire Hospitals NHS Foundation Trust, Crewe CW1 4QJ, UK; shingkwok@doctors.org.uk; 5 Division of Interventional Cardiology, Cardiovascular Diseases Department, University Hospital of Split, 21000 Split, Croatia

**Keywords:** coronary artery disease, coronary angiography, prevalence, renal artery stenosis, risk factors

## Abstract

**Background/Objectives**: Renal artery stenosis (RAS) is associated with coronary artery disease (CAD), exacerbation of arterial hypertension, and progression to heart failure, but remains frequently unrecognized in clinical practice. **Methods**: We conducted a systematic review and meta-analysis of studies by pooling data of patients undergoing CAG due to suspected or stable CAD that received a bilateral renal artery angiography. Results: A total of 31 studies with 31,689 patients were included (mean age 63.2 ± 8.7 years, 20.9% were female). Overall, 13.4% (95%CI 10.5–16.7%) of patients undergoing coronary angiography had significant RAS, with 6.5% (95% CI 4.5–8.9%) and 3.7% (95%CI 2.5–5.2%) having severe and bilateral RAS. The mean weighted proportion of patients with three-vessel coronary disease (3VD) was 25.1 (95%CI 19.6–30.9%) while 4.2% (95%CI 2.6–6.2%) had left main (LM) coronary disease. Patients with RAS compared to those without RAS were significantly older (mean difference, MD 4.2 years (95%CI 3.8–4.6)). The relative risk of RAS was greater for the female sex (risk ratio, 95%CI; RR 1.3, 1.03–1.57), presence of diabetes (RR 1.2, 1.10–1.36), arterial hypertension (RR 1.3, 1.21–1.46), dyslipidemia (RR 1.1, 1.06–1.14), peripheral artery disease (PAD) (RR 2.1, 1.40–3.16), chronic kidney disease (CKD) (RR 2.6, 2.04–3.37), 3VD (RR 1.6, 1.30–1.87), and LM disease (RR 1.8, 1.28–2.47). Smoking had a neutral effect on the risk of RAS occurrence (RR 1.0, 0.94–1.06). **Conclusions**: RAS is common in patients undergoing coronary angiography. CKD, PAD, older age, and severe CAD were among the strongest predictors for the presence of significant RAS.

## 1. Introduction

Renal artery stenosis (RAS) is an important but frequently unrecognized clinical condition. It shares common etiopathogenesis with other atherosclerotic diseases such as coronary artery disease (CAD), cerebrovascular disease (CVD), or peripheral artery disease (PAD) [1]. The detection of significant RAS is clinically relevant as the condition can perpetuate the progression of associated cardiovascular disease due to renovascular hypertension and lead to heart failure due to cardiorenal syndrome with renin–angiotensin–aldosterone (RAAS) system activation [2]. Among patients with suspected CAD undergoing diagnostic coronary angiography, presence of RAS carries important prognostic implications. It was independently associated with a 2-fold increased risk of all-cause mortality, regardless of many confounders and type of revascularization received [3]. Four-year survival among patients undergoing catheterization was 21% lower among patients with established RAS compared to those without RAS [4]. In patients with renal insufficiency or PAD, RAS was common, and its presence was strongly associated with increased mortality [5,6]. Even in asymptomatic individuals without known CVD, presence of renal artery calcification on CT was associated with increased all-cause mortality [7].

To our best knowledge, this study is the first systematic review and meta-analysis examining the prevalence and clinical factors associated with renal artery stenosis among patients with suspected or established CAD. We conducted an up-to-date analysis of the most relevant literature in past two decades that reported on both coronary and renal artery atherosclerosis in patients that underwent invasive workup.

Our primary objective was to determine and report on the prevalence of significant, severe, and bilateral RAS in this patient cohort. Secondary objectives were to determine the severity of coronary disease and to determine which clinical factors were associated with a risk of having RAS on angiography.

## 2. Materials and Methods

The search strategy was devised by one of the investigators (JAB), while the search of electronic databases was independently carried out by JAB and ISB. Electronic databases included in the search were the National Library of Medicine (NLM): PubMed, Ovid MEDLINE, Ovid Journals (full text), EMBASE, and SCOPUS. Search was conducted by using the following search terms: “*renal artery stenosis*” AND “*coronary artery disease*” AND “*diagnostic angiography*” AND/OR “*cardiac catheterization*”. These databases were manually searched to obtain full records of original articles (observational cohort studies) that were specifically designed to investigate and to report on the occurrence of renal artery stenosis in the setting of cardiac catheterization for stable CAD or suspected CAD. The search was limited to records published in relevant peer-reviewed journals in the English language in the last 20 years (from 2002 until 2022). Similarly, only observational cohort studies involving adult human subjects were considered. The date of the last database search was performed on 1 July 2022. All searches were independently manually performed by two reviewers (JAB and ISB), and potential studies were independently screened. Each investigator performed independent deletion of duplicate records, screening of available titles and abstracts, and provided final classification of studies as “*excluded*” or requiring further assessment or additional clarification. Such studies were labeled as “*potential for inclusion*”. If there was a discrepancy between the two investigators concerning the search strategy, this was resolved by the joint discussion involving the opinion of a third investigator (KS). The meta-analysis was conducted in accordance with the PRISMA reporting recommendations (Preferred Reporting Items for Systematic Review and Meta-Analyses) [8]. 

This study’s inclusion criteria were as follows: (a) adult patients, 18 years of age or older; (b) patients with established stable CAD (chronic coronary syndrome) or suspected CAD undergoing diagnostic coronary angiography accompanied with additional angiography of renal arteries; (c) non-randomized/retrospective/observational study design; and (d) studies explicitly reporting on the main outcome for this study which was the prevalence of renal artery stenosis in previously described cohorts. The exclusion criteria were as follows: (a) studies involving non-adult patients; (b) studies that examined renal artery stenosis in the setting of acute coronary syndromes; (c) studies designed as randomized controlled trials; (d) studies that were conducted among patients with established or suspected CAD undergoing diagnostic coronary angiography but did not perform concomitant angiography of renal arteries; (e) studies that did not report on the principal outcome of the interest such as the prevalence of renal artery stenosis (being significant and/or severe and/or bilateral); (f) studies that did not provide basic data on study length, setting, and provided no description about relevant baseline patients characteristics such as age, sex, comorbidities, and other clinical factors; and (g) studies that were duplicate reports without additional or updated outcome data.

The data were manually extracted by three investigators (JAB, ISB, and KS) and were inserted in predefined and customized tables in MS Word format. Baseline data on age, sex distribution, diabetes mellitus, arterial hypertension, dyslipidemia, smoking, renal failure, peripheral vascular disease, carotid artery stenosis, and previous myocardial infarction were captured in these tables. Furthermore, study data including the total number of enrolled patients, study period and location, type of study (multicentric or single-center), and study design were recorded. For each study, following angiographic variables and their prevalences were recorded in the predefined tables: significant RAS (in most studies defined as 50% or more luminal stenosis of at least one renal artery), severe RAS (in most studies defined as 70% or more luminal stenosis of at least one renal artery), bilateral RAS (significant RAS affecting both renal arteries), one-vessel CAD, two-vessel CAD, three-vessel CAD, and left main CAD. A quality assessment of the non-randomized studies was performed by using the Ottawa–Newcastle Scale [9]. All studies were independently scored by investigators KS and WM, and a maximum of 9 stars could be assigned to an individual study (shown in Supplemental Table S1).

For the estimation of prevalence of significant RAS, severe RAS, bilateral RAS, three-vessel CAD, and left main CAD, we used a weighted-proportion analysis, and for these endpoints, sample-size-weighted pooled proportions were reported at all instances. Risk ratio (RR) with 95% confidence intervals (95% CI) was used as the main summary measure for effect estimates on predefined dichotomous outcomes. A mean difference analysis was performed to determine possible absolute numerical differences in prespecified continuous outcomes such as age in patients with vs. without RAS. A random-effects model with Mantel–Haenszel statistics was applied for the principal meta-analysis. The meta-analysis was performed by using Review Manager software (RevMan, version 5.4, The Cochrane Collaboration, 2020) and MedCalc Statistical software (version 20.112, Medcalc Software Ltd., Ostend, Belgium).

A chi-square (χ^2^) test of heterogeneity and the Higgins *I*^2^ statistic of non-consistency were used to assess heterogeneity across the included studies. Studies with an *I*^2^ statistic of 15% to <35% were considered to have low heterogeneity, studies with an *I*^2^ statistic of >35% to 75% were considered to have moderate heterogeneity, and studies with an *I*^2^ statistic of >75% were considered to have a high heterogeneity.

Publication bias was assessed by a visual inspection of obtained funnel plots and with a formal Egger’s test calculation. In this regard, *p*-values < 0.05 indicated significant publication bias across included studies. All *p*-values reported in this manuscript were two-tailed, and the results were considered statistically significant if *p* < 0.05 unless explicitly stated otherwise.

## 3. Results

**Figure 1:**
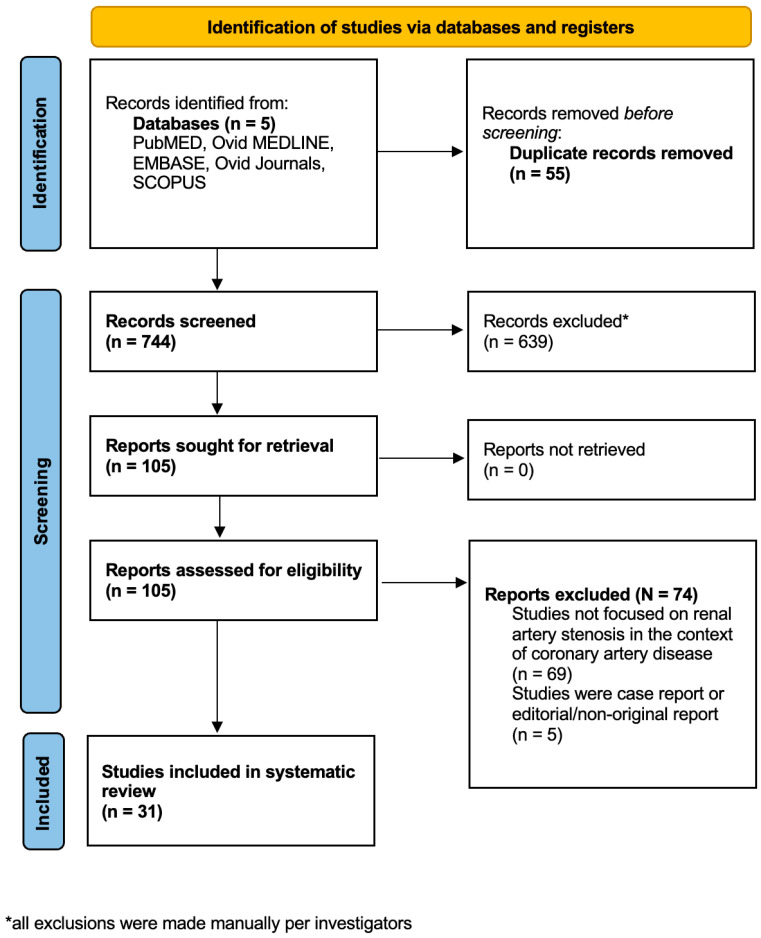
PRISMA flowchart depicting selection and inclusion process of potential studies.

Thirty-one international studies with total of 31,689 patients enrolled were included in the final analysis, as shown in the PRISMA flowchart depicted in Figure 1 [10,11,12,13,14,15,16,17,18,19,20,21,22,23,24,25,26,27,28,29,30,31,32,33,34,35,36,37,38,39,40].

Most studies were single-center, prospective, or observational cohort studies. Only two were multicenter cross-sectional studies [16,32]. Three studies were from North America, six from Europe, seven from Central or East Asia, thirteen from Middle East or North Africa, and two from South America as shown in Table 1.

The weighted mean age of the entire studied population was 63.2 ± 8.7 years. Across the whole patient sample, the women were represented with a weighted average of 36.4%. The weighted mean proportion of standard modifiable cardiovascular risk factors such as diabetes mellitus, arterial hypertension, dyslipidemia, smoking, peripheral vascular disease, carotid artery stenosis, and history of MI are shown in Table 2 while detailed baseline patient characteristics across individual studies are provided in Supplemental Table S1.

**Figure 2:**
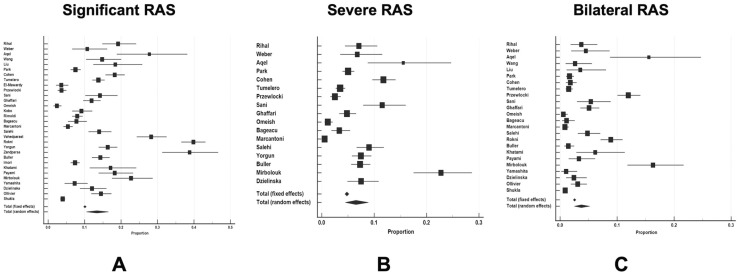
The pooled weighted proportions of significant, severe, and bilateral renal artery stenosis (RAS). Panel (**A**), proportion of significant RAS; Panel (**B**), proportion of severe RAS; Panel (**C**), proportion of bilateral RAS in patients undergoing simultaneous coronary artery and renal artery angiography. **Abbreviations**: RAS—renal artery stenosis.

The pooled proportion of significant RAS (≥50% stenosis) in 22,757 patients undergoing coronary angiography was 13.4% (95% CI 10.5–16.7%) as shown in Figure 2A. Furthermore, the pooled proportion of severe RAS (≥70% stenosis) in 11,903 patients undergoing coronary angiography was 6.5% (95% CI 4.5–8.9%) as shown in Figure 2B. Finally, the pooled proportion of bilateral RAS in 17,167 patients undergoing coronary angiography was 3.7% (95% CI 2.5–5.2%) as depicted in Figure 2C.

**Figure 3:**
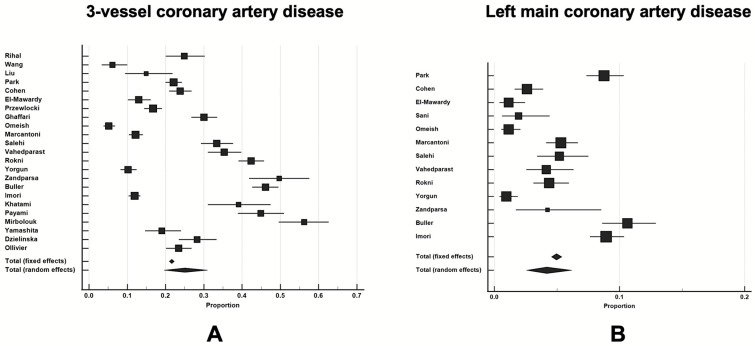
The pooled weighted proportions of three-vessel disease: Panel (**A**), based on available data from 14,771 patients and left main disease; Panel (**B**), based on available data from 10,670 patients with CAD undergoing cardiac catheterization.

In terms of angiographic coronary artery disease (CAD) burden, the pooled weight-adjusted proportion of three-vessel coronary artery disease (3VD) was 25.1 (95% CI 19.6–30.9%), and this finding was based on data from 14,771 patients pooled from 23 studies (Figure 3A). Significant left main (LM) coronary disease was detected in 4.2% of cases (95% CI 2.6–6.2%), and this was based on data from 10,670 patients from 13 studies (Figure 3B). Detailed angiographic characteristics for each particular study (where available) are also provided in Supplemental Table S2. Detailed characteristics of each particular study included in this systematic review and meta-analysis are shown in Supplemental Table S3.

The impact of anthropometric factors such as age and female sex and cardiovascular/clinical factors including diabetes mellitus (DM), arterial hypertension, dyslipidemia, smoking, chronic kidney disease (CKD), three-vessel coronary disease (3VD), left main (LM) disease, and peripheral artery disease (PAD) were evaluated for the potential association with RAS occurrence in the meta-analysis. As shown in Table 3 and in the order of decreasing magnitude, CKD was found to be the most robust predictor of RAS as it was associated with a more than 2.5-fold increase in the relative risk of RAS compared to patients without CKD. This was followed by PAD and LM disease that were associated with a 2-fold and 1.8-fold increases in the relative risk of RAS occurrence. Likewise, 3VD was associated with a 56% relative risk increase in RAS.

Female sex and arterial hypertension were both associated with an increase in a relative risk of RAS (RR 1.3 and RR 1.33,) while smoking was not identified as a significant variable impacting on RAS occurrence (RR 1.0). Similarly, diabetes mellitus and dyslipidemia were associated with 22% and 10% increases in the relative risk of RAS occurrence (RR 1.2 and RR 1.10). In terms of age, the meta-analysis showed that patients with RAS were significantly older than patients without RAS. In fact, there was a mean age difference of 4.16 years (95% CI 3.75–4.58 years) between patients with RAS versus those without RAS. 

Detailed analyses with generated visual funnel plots (publication bias) and forest plots of individual clinical risk factors and consequent risk ratios comparing patients with RAS vs. non-RAS are provided in Supplementary Materials (Supplemental Figures S1–S15).

## 4. Discussion

We present the largest published data set on patients undergoing simultaneous coronary artery and renal artery catheterization. The key finding of our systematic review and meta-analysis is that among patients undergoing coronary angiography, around 13% have a significant RAS. About 6% of patients will have severe RAS, and in almost 4% of patients, RAS is bilateral. Most included studies reported a similar prevalence; however, two papers reported a markedly higher prevalence of significant RAS (38 to 39%) [30,40]. Part of the reasons why in these two studies the proportions of RAS were higher could be due to the pooling and reporting of significant and severe RAS together. Another reason is possibly a high proportion of patients with previous MI (36%) in a study by Rokni et al. which is in contrast to other studies (range 15–30%) and hence possibly indicating a population with higher end-organ damage [30]. Both studies originate from Iran and therefore racial or regional factors may play an additional role.

The prevalence of significant RAS in patients undergoing coronary angiography appears high compared to previously reported 2.7% found among patients with moderate-to-severe arterial hypertension [41]. In a population with established end-organ disease, such as peripheral artery disease (PAD), the prevalence of significant RAS was with 9.6% similar to our findings [42]. In very high-risk cohorts, RAS was previously even more frequently reported. Kuroda et al. described in a small study of patients who suffered fatal stroke on autopsy severe RAS (≥75% luminal area narrowing) in 10.4% of patients, and the proportion of severe RAS was even higher in a subgroup of stroke patients who died and were known to have renal insufficiency with 28.6% [43]. These data reflect intricate pathophysiological relationships since significant or severe renal artery stenosis is an important driver of renal failure and CKD, while CKD is an independent risk factor for CAD development.

Another key finding of our study was the determination of clinical factors associated with RAS occurrence. Factors showing the strongest association were older age, known CKD, and PAD. Patients with RAS were more than 4 years older compared to patients without RAS. Pre-existing CKD increased the relative risk of having RAS by almost three-fold, while patients with PAD were almost 2.5 times more at risk of having a significant RAS. Other clinical factors increasing the relative risk of RAS by 20 to 90% were female sex, arterial hypertension, diabetes, 3VD, and LM disease. Interestingly, smoking did not pose any additional risk. However, it should be noted that smoking was not equally defined in all studies as it is unclear if some studies considered previous smoking as smoking or if only active smoking at the time of enrollment was captured. Other authors reported similar findings. For example, Ozkan et al. showed that in PAD patients, advanced age and hypertension were closely associated with the presence of significant RAS [42]. Sani et al. reported in 260 hypertensive and/or diabetic consecutive patients who underwent simultaneous coronary and renal artery catheterization that female sex, multivessel coronary disease, and a reduced glomerular filtration rate were independent predictors of significant RAS [32]. In a smaller study (n = 165), Zandparsa et al. again identified arterial hypertension and increased serum creatinine levels as independent predictors of RAS. However, in this study, the severity of CAD as assessed by the Gensini score was not predictive of RAS [44]. This is possibly due to the rather smaller sample size.

The important question remaining is the clinical implication of RAS finding during coronary angiography and if such investigation should be pursued: how this finding can influence future diagnostic and therapeutic strategies or, in other words, if the RAS is detected, whether a simultaneous percutaneous coronary intervention (PCI) and intervention on renal arteries is feasible and what the clinical impact and *net* potential benefit would be for the patient. Unfortunately, there is a great paucity of data on this topic. Dong et al. published an article describing a retrospective cohort of 149 patients with simultaneous PCI and PTRAS (percutaneous transluminal renal artery stenting) and reported on the feasibility and safety of such an approach. This intervention led to improved arterial blood pressure control and a reduced left ventricular mass (LVM) index; however, it did not impact changes in renal function [45]. Reznik et al. reported similar finding of LVM reduction in patients undergoing RAS stenting; however, in their study, this effect was independent from blood pressure reduction [46]. Following RAS stenting, reduction in LV filling pressures was observed in heart failure (HF) patients [47]. However, no randomized studies have thus far addressed the approach of PCI + PTRAS vs. PCI alone in patients with both established CAD and RAS and if such an approach would improve clinical and patient-oriented outcomes. The retrospective study of Dong and colleagues combining PCI and PTRAS suggested a *net* clinical benefit in population of patients with HF and preserved ejection fraction; however, these findings should be first replicated in randomized studies.

Taken together, such a dual revascularization approach might be feasible for selected patient populations exhibiting both high-risk coronary and renal anatomy, thus reflecting severe atherosclerotic burden. The risk of contrast-induced nephropathy (CIN) should be weighed against the potential benefits of dual revascularization and even vascular access approach and other factors might play an important role in this—e.g., a femoral vs. radial approach (femoral naturally being more convenient for performing bilateral renal angiography) and the experience of the operator (more experienced operators will likely use fewer contrast injections and will establish faster access to designated vascular territories).

There are several limitations to our study. First, the exact technique of RAS severity assessment is not described in detail in many of the studies involved in this analysis. Secondly, many of the studies do not describe clearly which cases of bilateral RAS had significant or severe stenoses. Third, the lack of individual data does not allow a direct comparison between patients who had normal coronary angiograms vs. patients who had significant coronary artery disease. However, pooled risk ratios from individual analyses confirm a significantly higher proportion of RAS occurrence in patients with significant coronary disease burden. Furthermore, due to the observational design of studies included in the analysis and the lack of consecutive enrollment of patients in most of the studies, a potential *selection bias* should be acknowledged. In that sense, it remains unclear how and which patients were selected to receive renal artery angiography on top of standard coronary angiography. An issue of high heterogeneity for some of the outcomes should be mentioned, which is likely to be explained by the large variability of studies in terms of their country of origin, patient population, regional healthcare practices, and differences in definition/reporting/diagnostic criteria of the RAS. It is worth noting that most of the included studies were single-center and from specific geographical regions which might be perceived as a limit; however, we included a robust number of studies from various centers worldwide which should contribute to study generalizability and representativeness of the presented findings. Some of the studies predate the era of coronary CT, and hence, the selection of patients presenting to cardiac laboratory is nowadays possibly affected, and patients with systemic atherosclerosis could be better preselected by CTs in the modern era. Our study does not imply that CAD itself is a predictor of RAS. Rather, our results describe the impact of traditional CAD risk factors on the prevalence of RAS diagnosis.

## 5. Conclusions

Renal artery stenosis is common in patients undergoing coronary artery catheterization. Our results show that between 1:7 and 1:10 of all-comers undergoing diagnostic coronary angiography are likely to have significant RAS. Clinical factors such as chronic kidney disease, peripheral artery disease, older age, and severe coronary artery disease are the strongest associated factors with risk of having a significant RAS. Factors showing weaker but significant association with significant RAS were female gender, arterial hypertension, dyslipidemia, and diabetes mellitus. Smoking does not appear to be associated with RAS. Future prospective studies should be oriented to determine the importance of early RAS detection in high-risk populations.

## Figures and Tables

**Table 1:** The overview of the design and setting of studies included in the analysis.

Authors of the Study and Year	Ref.	Total Number of Patients	Study Period	Study Location	Multicenter or Single-Center Study	Study Type
Rihal et al., 2002	[28]	N = 300	July 1998 to March 1999	Mayo Clinic, Rochester, USA	Single-center	Prospective cohort analysis
Weber-Mzell et al., 2002	[37]	N = 177	-	University Graz, Austria	Single-center	Cohort study
Yamashita et al., 2002	[38]	N = 289	April 2000 to October 2000	Kitami Red Cross Hospital, Japan	Single-center	Cohort study
Aqel et al., 2003	[10]	N = 542	February 2001 to November 2001	Veterans’ Administration (VA) Medical Center, USA	Single-center	Prospective study
Wang et al., 2003	[36]	N = 230	-	Queen Mary Hospital, Hong Kong	Single-center	Prospective study
Liu et al., 2004	[20]	N = 141	January 2000 to March 2004	Zhong Da Hospital, Nanjing, PR China	Single-center	Cohort study
Park et al., 2004	[25]	N = 1459	March 1998 to July 1999	Yonsei University Cardiovascular Center, Seoul, South Korea	Single-center	Retrospective cohort study
Cohen et al., 2005	[13]	N = 843	September 2000 to May 2002	Hospital Italiano de Buenos Aires, Argentina	Single-center	Prospective study
Dzielinska et al., 2007	[14]	N = 333	-	Institute of Cardiology in Warsaw, Poland	Single-center	Prospective cohort study
Tumelero et al., 2006	[34]	N = 1656	January 2002 to February 2004	Hospital Sao Vicente de Paulo, Passo Fondo, Brazil	Single-center	Prospective cross-sectional study
Ollivier et al., 2009	[23]	N = 650	May 2004 to May 2006	CHU de Rennes, France	Single-center	Prospective cohort study
El-Mawardy et al., 2008	[15]	N = 525	November 2000 to June 2002	Ain Shams University Hospital, Cairo, Egypt	Single-center	Cohort study
Przewlocki et al., 2008	[27]	N = 1036	Period of 12 months	University Hospital, Krakow, Poland	Single-center	Cohort study
Sani et al., 2008	[32]	N = 260	April 2005 to 2006	Two educational hospitals in Mashhad (Emam Reza and Qaem), Iran	Multicenter	Cross-sectional study
Ghaffari et al., 2009	[16]	N = 732	April 2007 to May 2008	3 hospitals in Tabriz, Iran	Multicenter	Cross-sectional study
Omeish et al., 2009	[24]	N = 870	January 2006 to April 2006	Queen Alia Heart Institute, Amman, Jordan	Single-center	Prospective cross-sectional study
Kobo et al., 2010	[19]	N = 7500	2001 to 2007	Bnai-Zion Medical Center, Haifa, Israel	Single-center	Cohort study
Rimoldi et al., 2010	[29]	N = 1504	1st of January 2004 to 31st of August 2007	Swiss Cardiovascular Center Bern, University Hospital Bern, Bern, Switzerland	Single-center	Retrospective study
Bageacu et al., 2011	[11]	N = 492	4-month period	University Hospital of Saint-Erienne, France	Single-center	Prospective study
Marcantoni et al., 2013	[21]	N = 1298	April 2007 to March 2008	The Division of Cardiology, University of Catania, Italy	Single-center	Prospective study
Salehi et al., 2011	[31]	N = 500	Period of 12 months from November 2008	Shaheed Rajeie Cardiovascular Medical, and Research Center, Iran	Single-center	Prospective cohort study
Vahedparast et al., 2011	[35]	N = 835	August 2008 to August 2009	Bent Al-Hoda Hospital od Bushehr University of Medical Science, Iran	Single-center	Prospective cross-sectional study
Rokni et al., 2012	[30]	N = 18,419	October 2009 to July 2011	Tehran Heart Center, Iran	Single-center	Retrospective cross-sectional study
Yorgun et al., 2013	[39]	N = 832	-	Hacettepe University, Ankara, Turkey	Single-center	Observational study
Zandparsa et al., 2012	[40]	N = 165	September 2010 to May 2011	Tehran University of Medical Sciences, Tehran, Iran	Single-center	Cohort study
Shukla et al., 2013	[33]	N = 3500	January 2012 to June 2012	Civil Hospital, Ahmedabad, India	Single-center	Prospective cohort study
Buller et al., 2004	[12]	N = 851	June 2001 to May 2002	Vancouver Hospital, Canada	Single-center	Prospective cohort study
Imori et al., 2014	[17]	N = 2571	September 2010 to July 2011	Shonan Kamakura General Hospital, Kanagawa, Japan	Single-center	Cross-sectional analysis
Khatami et al., 2014	[18]	N = 173	-	Tehran University of Medical Sciences, Tehran, Iran	Single-center	Cross-sectional study
Payami et al., 2016	[26]	N = 312	March 2009 to October 2010	Emam Hospital, Ahvaz, Iran	Single-center	Cross-sectional study
Mirbolouk et al., 2019	[22]	N = 247	May 2015 to June 2016	Heshmat Heart Hospital, Rasht, Iran	Single-center	Cross-sectional study

**Table 2:** Pooled weighted proportions (%) and mean of baseline patient characteristics from included studies.

Variable	Mean ± SD or % (95% CI)
Age, mean (years)	63.2 ± 8.7
Female sex, %	36.4 (32.4–40.5)
Diabetes mellitus, %	28.7 (25.0–32.5)
Arterial hypertension, %	80.3 (70.3–88.6)
Dyslipidemia, %	61.6 (53.5–69.3)
Smoking, %	38.4 (31.7–45.2)
Renal failure, %	10.9 (7.0–15.6)
Peripheral vascular disease, %	14.9 (6.9–25.2)
Carotid artery disease, %	23.4 (2.9–55.4)
Previous myocardial infarction, %	22.9 (18.7–27.4)

Abbreviations: CI—confidence interval; SD—standard deviation.

**Table 3:** Clinical factors associated with the risk of RAS occurrence during coronary angiography in CAD patients.

Variable	Risk Ratio (RR)	95% Confidence Interval	*p*-Value	Heterogeneity *
**Female sex** N = 27 studies	1.27	1.03–1.57	0.030	High *I*^2^ = 92%
**Diabetes mellitus** N = 28 studies	1.22	1.10–1.36	<0.001	Moderate *I*^2^ = 57%
**Arterial hypertension** N = 19 studies	1.33	1.21–1.46	<0.001	High *I*^2^ = 94%
**Dyslipidemia** N = 24 studies	1.10	1.06–1.14	<0.001	Moderate *I*^2^ = 59%
**Current smoking** N = 24 studies	1.00	0.94–1.06	0.930	Low *I*^2^ = 26%
**Chronic kidney disease** N = 13 studies	2.62	2.04–3.37	<0.001	Moderate *I*^2^ = 66%
**Three-vessel disease** N = 17 studies	1.56	1.30–1.87	<0.001	High *I*^2^ = 81%
**Left main disease** N = 10 studies	1.78	1.28–2.47	<0.001	Moderate *I*^2^ = 52%
**Peripheral artery disease** N = 13 studies	2.11	1.40–3.16	<0.001	High *I*^2^ = 94%

* The heterogeneity of each meta-analysis was determined by a chi-square (χ^2^) test of heterogeneity and the Higgins *I*^2^ statistic, and the following criteria were applied: studies with an *I*^2^ statistic of 15% to <35% were considered to have low heterogeneity; those with an *I*^2^ statistic of >35% to 75% were considered to exhibit a moderate heterogeneity; and those with an *I*^2^ statistic of >75% were considered to exhibit a high heterogeneity.

## Data Availability

The data presented in this study are available upon reasonable request from the corresponding author.

## Supplementary Materials

The following supporting information can be downloaded at https://www.mdpi.com/article/10.3390/diseases12090208/s1, Supplemental Figures S1–S15: Publication bias blots and calculations for the pooled proportions; Table S1: Baseline demographic, comorbidity, and clinical characteristics of patients across studies included in the analysis; Table S2: Angiographic characteristics of renal artery stenosis (RAS) and coronary artery disease (CAD) across included studies; Table S3: Detailed characteristics of each individual study included in this systematic review and meta analysis; Table S4: Study quality assessment using the Ottawa-Newcastle Scale for observational/cohort studies.
