# Supplementary material for: Prevalence and Risk Factors of Renal Artery Stenosis in Patients Undergoing Simultaneous Coronary and Renal Artery Angiography: A Systematic Review and Meta-Analysis of 31,689 Patients from 31 Studies
Source: Diseases. 2024 Sep 11;12(9):208. doi: 10.3390/diseases12090208 (PMC11431457; doi:10.3390/diseases12090208)
Supplement: Supplementary file 1 [file diseases-12-00208-s001.zip › Supplementary Materials.pdf]

SUPPLEMENTAL APPENDIX

Supplemental figures - publication bias blots and calculations for the pooled proportions

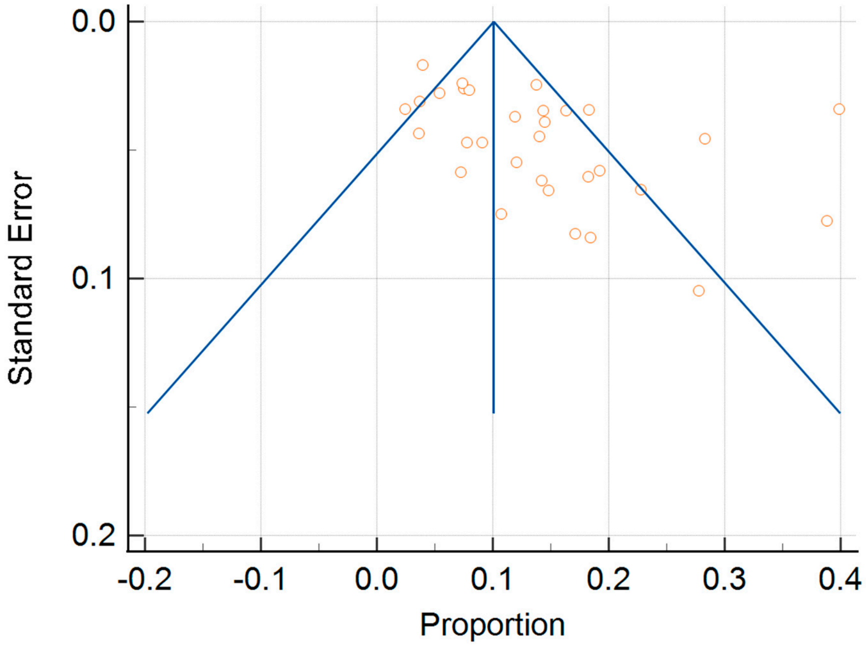

Supplemental Figure S1. Publication bias for the calculation of significant RAS proportion

The data for the pooled proportion of RAS (weight-adjusted for the size of the studies and defined as at least ≥ 50% stenosis in at least one of the renal arteries) were marked by high degree of heterogeneity (I^2=97.8%, P<0.001) with significant publication bias detected (Egger's test P=0.0059).

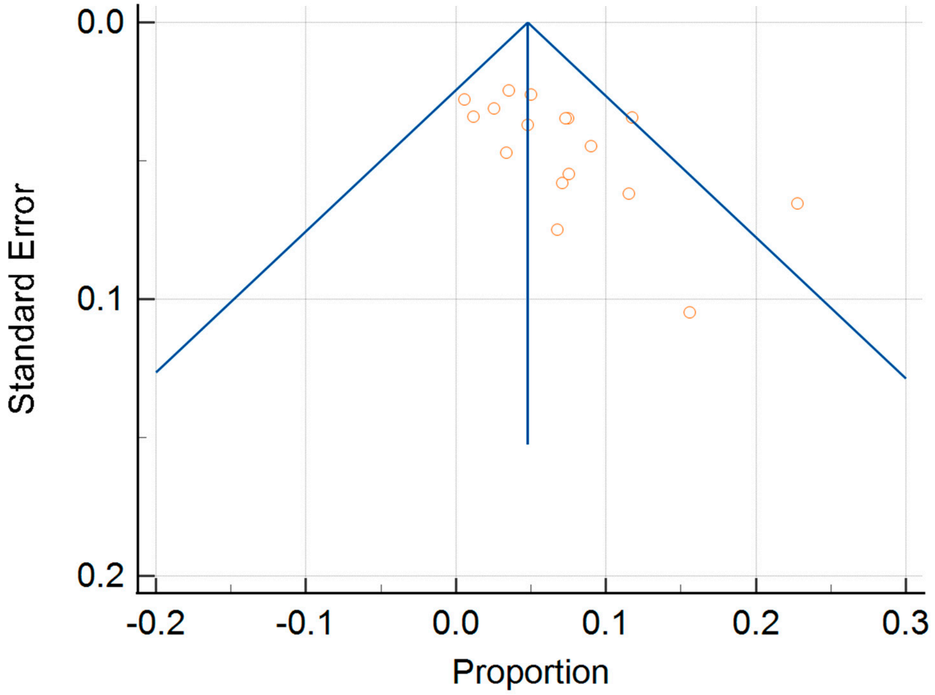

Supplemental Figure S2. Publication bias for the calculation of severe RAS proportion

Pooled proportion of severe RAS (weight-adjusted for the size of studies and defined as at least ≥70% stenosis in at least one of the renal arteries) in analyzed sample was of patients undergoing coronary angiography had significant RAS (>50% stenosis), 6.52% [4.48, 8.91] % ; mean [95% CI]. This finding was based on data from 11,903 patients pooled from 17 individual studies. The data were marked by high degree of heterogeneity (I^2=95.78%, P<0.001) while significant publication bias was also detected for this endpoint (Egger's test P=0.0205)

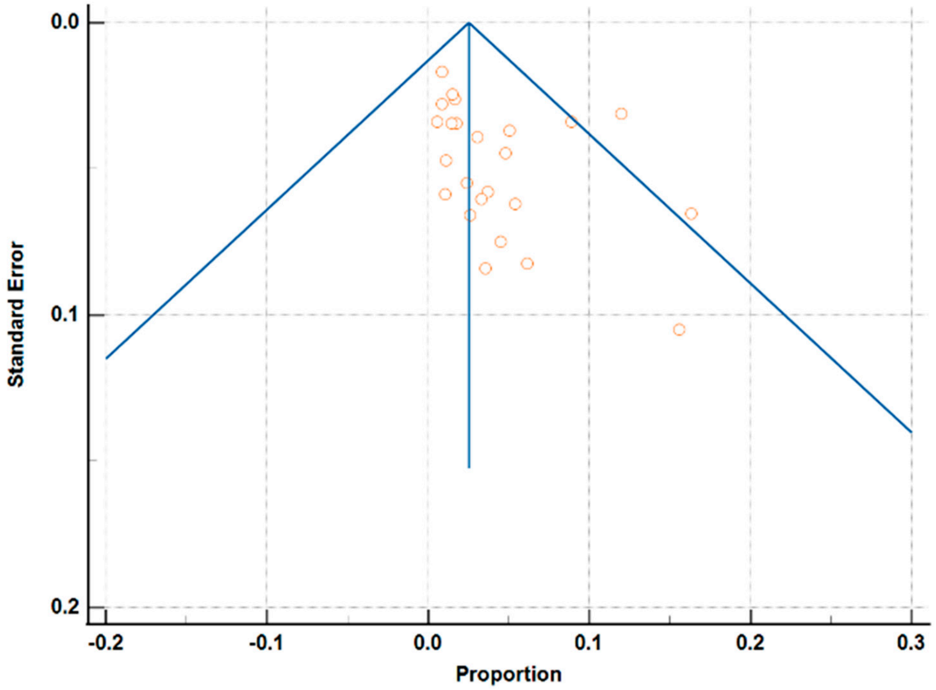

Supplemental Figure S3. Publication bias for the calculation of bilateral RAS proportion

Bilateral RAS was marked by the pooled weight-adjusted proportion of 3.72% [2.48, 5.19] %; mean [95% CI] while this finding was based on data from 17,167 patients pooled from 24 studies. A high degree of heterogeneity was established (I^2=95.28 %, P<0.001) while significant publication bias was also detected (Egger's test P=0.0210).

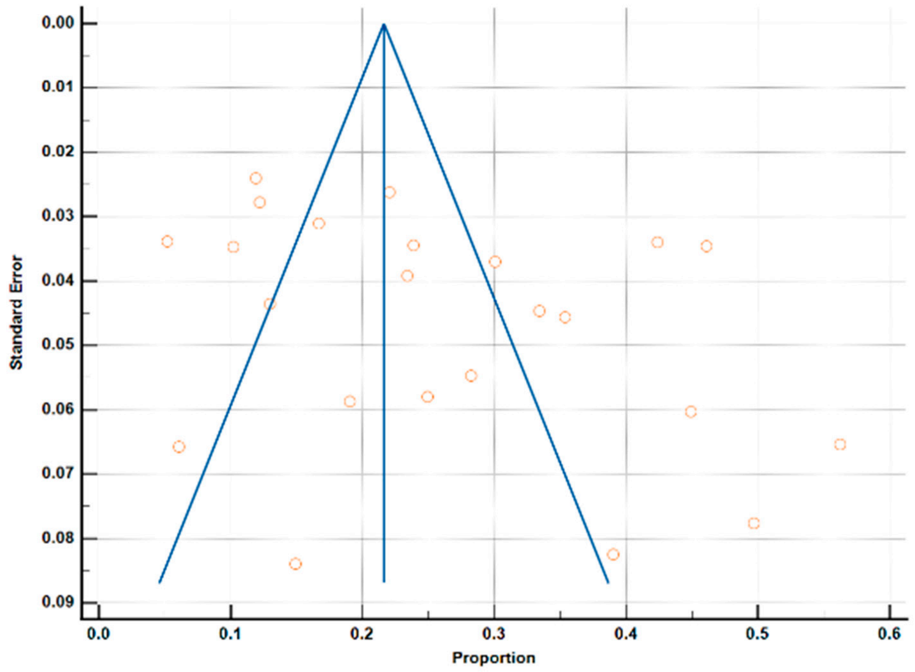

Supplemental Figure S4. Publication bias for the calculation of proportion of three-vessel coronary artery

The pooled weight-adjusted proportion of three-vessel coronary artery disease (3VD) was 25.06% (95% CI 19.61, 30.93] %, and this finding was based on data from 14,771 patients pooled from 23 studies. Data were marked by the high degree of heterogeneity (I^2=98.44%, P<0.001) while no signifiant publication bias was detected (Egger's test P=0.0807).

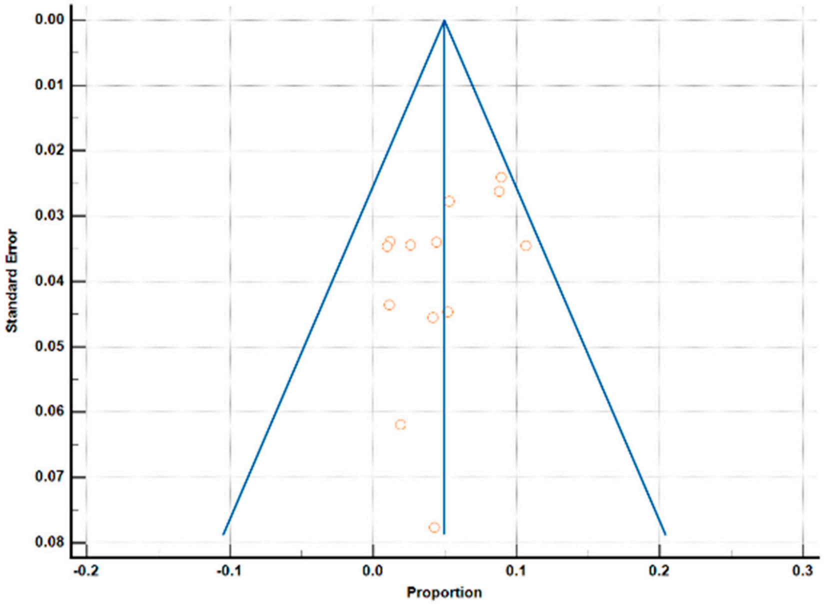

Supplemental Figure S5. Publication bias for the calculation of proportion of the left main disease

Significant left main (LM) disease was present among patients with significant RAS in 4.19% of cases [2.56, 6.18] % based on reports from 10,670 patients from 13 studies. This calculation was marked by the high degree heterogeneity (I^2=95.41%, p<0.001) while no significant publication bias was detected (Egger's test p=0.1241).

Forest plots of the analyses concerning individual clinical factors for RAS occurence

AGE

Study or Subgroup	RAS Mean	RAS SD	RAS Total	No RAS Mean	No RAS SD	No RAS Total	Weight	Mean Difference IV, Fixed, 95% CI	Year
Rihal 2002	67	11	57	64	10	240	1.8%	3.00 [−0.12, 6.12]	2002
Weber 2002	67	8	19	61	11	158	1.1%	6.00 [2.01, 9.99]	2002
Yamashita 2002	68.9	8.7	21	65.6	10.8	268	1.1%	3.30 [−0.64, 7.24]	2002
Aqel 2003	63.7	9.1	25	60.7	8.7	65	1.0%	3.00 [−1.15, 7.15]	2003
Wang 2003	70.6	6.3	34	64.2	7.8	196	3.1%	6.40 [4.02, 8.78]	2003
Liu 2004	63	13	26	54	6	115	0.7%	9.00 [3.88, 14.12]	2004
Park 2004	63.2	8.5	158	59.2	9.9	1301	8.5%	4.00 [2.57, 5.43]	2004
Cohen 2005	68.5	7.5	253	63	8	689	14.3%	5.50 [4.40, 6.60]	2005
Tumelero 2006	66.4	11.6	228	61.5	29.4	1428	3.8%	4.90 [2.76, 7.04]	2006
Dzielinska 2007	59.8	9.6	40	56.6	9.5	293	1.7%	3.20 [0.03, 6.37]	2007
El−Mawardy 2008	53.8	11.6	19	53.2	9.5	506	0.6%	0.60 [−4.68, 5.88]	2008
Przewlocki 2008	0	0	0	0	0	0		Not estimable	2008
Sani 2008	62.1	10	37	56.3	8.9	223	1.5%	5.80 [2.37, 9.23]	2008
Ghaffari 2009	58.8	8.7	87	59.5	9.8	645	4.4%	−0.70 [−2.68, 1.28]	2009
Omeish 2009	68	8	21	61	11	849	1.4%	7.00 [3.50, 10.50]	2009
Ollivier 2009	69	10	94	67	10	556	3.6%	2.00 [−0.19, 4.19]	2009
Kobo 2010	70	9	41	63.5	10.3	409	2.0%	6.50 [3.57, 9.43]	2010
Rimoldi 2010	69.5	9	112	64.5	10	1291	5.6%	5.00 [3.25, 6.75]	2010
Bageacu 2011	0	0	0	0	0	0		Not estimable	2011
Marcantoni 2011	68	8	70	63	10	1228	4.5%	5.00 [3.04, 6.96]	2011
Salehi 2011	63.1	8.7	70	59.5	9.9	430	3.5%	3.60 [1.36, 5.84]	2011
Vahedparast 2011	62.7	10.8	136	57.9	10.5	345	3.8%	4.80 [2.67, 6.93]	2011
Rokni 2012	66.2	9.7	345	61.6	10.3	521	9.5%	4.60 [3.25, 5.95]	2012
Yorgun 2012	65.5	10.3	136	60.4	8.8	696	5.1%	5.10 [3.25, 6.95]	2012
Zandparsa 2012	60.4	9.6	64	59.5	9.5	101	1.9%	0.90 [−2.09, 3.89]	2012
Shukla 2013	58.8	11.5	139	59.5	10.8	61	1.6%	−0.70 [−4.02, 2.62]	2013
Buller 2014	71.2	7.6	120	67.2	9.6	717	7.4%	4.00 [2.47, 5.53]	2014
Imori 2014	0	0	0	0	0	0		Not estimable	2014
Khatami 2014	65.4	8.6	37	59.8	9.4	109	1.6%	5.60 [2.31, 8.89]	2014
Payami 2016	64	10.1	50	59.8	11.1	224	1.7%	4.20 [1.05, 7.35]	2016
Mirbolouk 2019	64.5	8	76	64	10	157	3.1%	0.50 [−1.88, 2.88]	2019
**Total (95% CI)**			**2515**			**13821**	**100.0%**	**4.16 [3.75, 4.58]**	

Heterogeneity: $Chi^2 = 75.97$, $df = 27$ ($P < 0.00001$); $I^2 = 64\%$

Test for overall effect: $Z = 19.59$ ($P < 0.00001$)

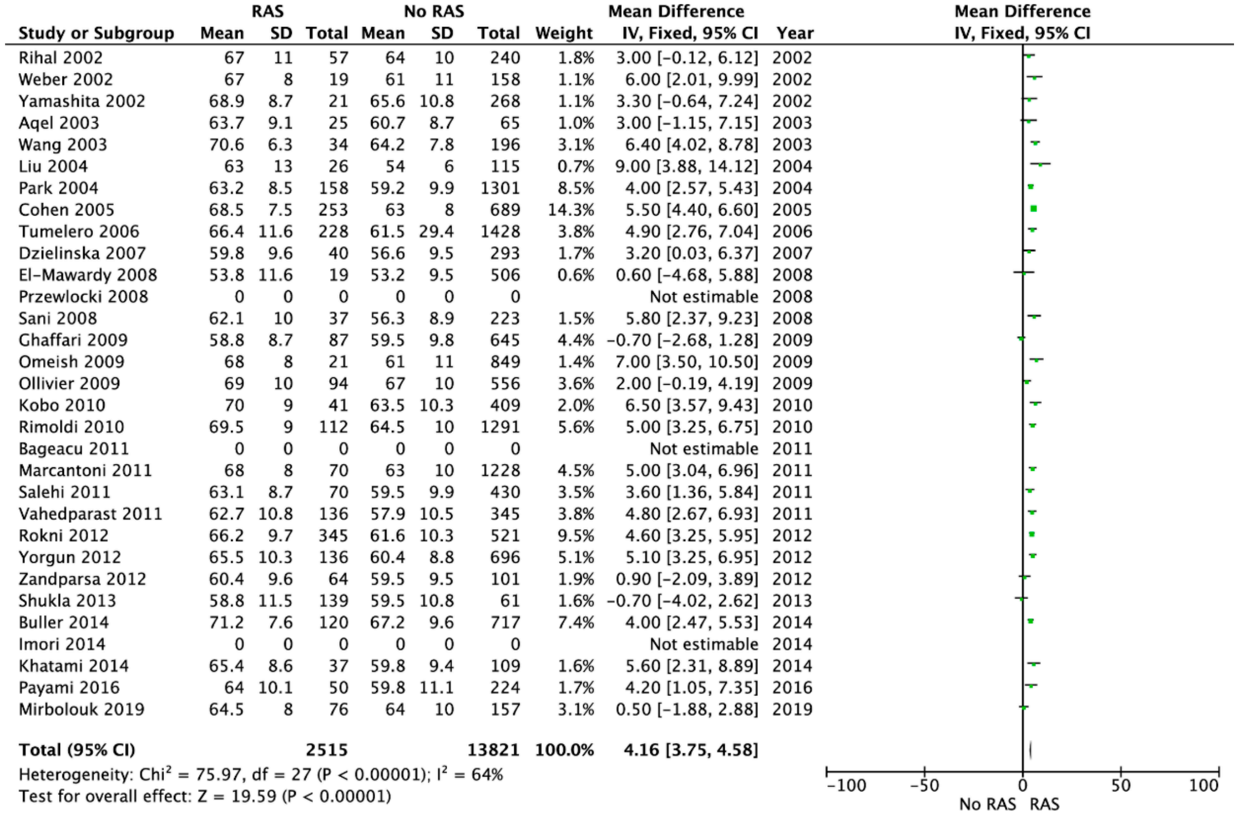

Supplemental Figure S6. Mean difference in age with respect if patients had RAS or did not have RAS

FEMALE SEX

Study or Subgroup	RAS Events	RAS Total	No RAS Events	No RAS Total	Weight	Risk Ratio M-H, Random, 95% CI	Year
Rihal 2002	0	0	0	0		Not estimable	2002
Weber 2002	1	19	49	158	0.9%	0.17 [0.02, 1.16]	2002
Yamashita 2002	10	21	114	268	3.6%	1.12 [0.70, 1.79]	2002
Wang 2003	18	34	64	196	3.9%	1.62 [1.11, 2.36]	2003
Aqel 2003	0	25	2	65	0.4%	0.51 [0.03, 10.22]	2003
Liu 2004	11	26	71	115	3.6%	0.69 [0.43, 1.10]	2004
Park 2004	0	0	0	0		Not estimable	2004
Cohen 2005	86	253	199	689	4.3%	1.18 [0.96, 1.45]	2005
Tumelero 2006	132	228	131	1428	4.3%	6.31 [5.18, 7.69]	2006
Dzielinska 2007	9	40	85	293	3.2%	0.78 [0.42, 1.42]	2007
El-Mawardy 2008	6	19	99	506	3.0%	1.61 [0.81, 3.20]	2008
Przewlocki 2008	0	0	0	0		Not estimable	2008
Sani 2008	25	37	100	223	4.1%	1.51 [1.15, 1.97]	2008
Ghaffari 2009	51	87	365	645	4.3%	1.04 [0.86, 1.25]	2009
Omeish 2009	12	21	272	849	3.9%	1.78 [1.22, 2.62]	2009
Ollivier 2009	18	94	119	556	3.7%	0.89 [0.57, 1.40]	2009
Kobo 2010	26	41	133	409	4.1%	1.95 [1.49, 2.56]	2010
Rimoldi 2010	47	112	595	1291	4.2%	0.91 [0.73, 1.14]	2010
Marcantoni 2011	27	70	344	1228	4.1%	1.38 [1.01, 1.88]	2011
Salehi 2011	35	70	201	430	4.2%	1.07 [0.83, 1.38]	2011
Vahedparast 2011	68	136	171	345	4.3%	1.01 [0.83, 1.23]	2011
Bageacu 2011	11	34	116	416	3.5%	1.16 [0.70, 1.93]	2011
Rokni 2012	162	345	210	521	4.3%	1.16 [1.00, 1.36]	2012
Yorgun 2012	28	136	150	696	3.9%	0.96 [0.67, 1.37]	2012
Zandparsa 2012	37	64	41	101	4.0%	1.42 [1.04, 1.95]	2012
Shukla 2013	36	139	18	61	3.6%	0.88 [0.54, 1.42]	2013
Imori 2014	0	0	0	0		Not estimable	2014
Khatami 2014	24	37	27	109	3.8%	2.62 [1.75, 3.92]	2014
Buller 2014	47	120	164	717	4.2%	1.71 [1.32, 2.22]	2014
Payami 2016	32	50	134	224	4.2%	1.07 [0.85, 1.35]	2016
Mirbolouk 2019	42	76	88	157	4.2%	0.99 [0.77, 1.26]	2019
**Total (95% CI)**		**2334**		**12696**	**100.0%**	**1.27 [1.03, 1.57]**	
Total events	1001		4062				

Heterogeneity: $Tau^2 = 0.26$; $Chi^2 = 333.78$, df = 26 ($P < 0.00001$); $I^2 = 92\%$
Test for overall effect: $Z = 2.24$ ($P = 0.03$)

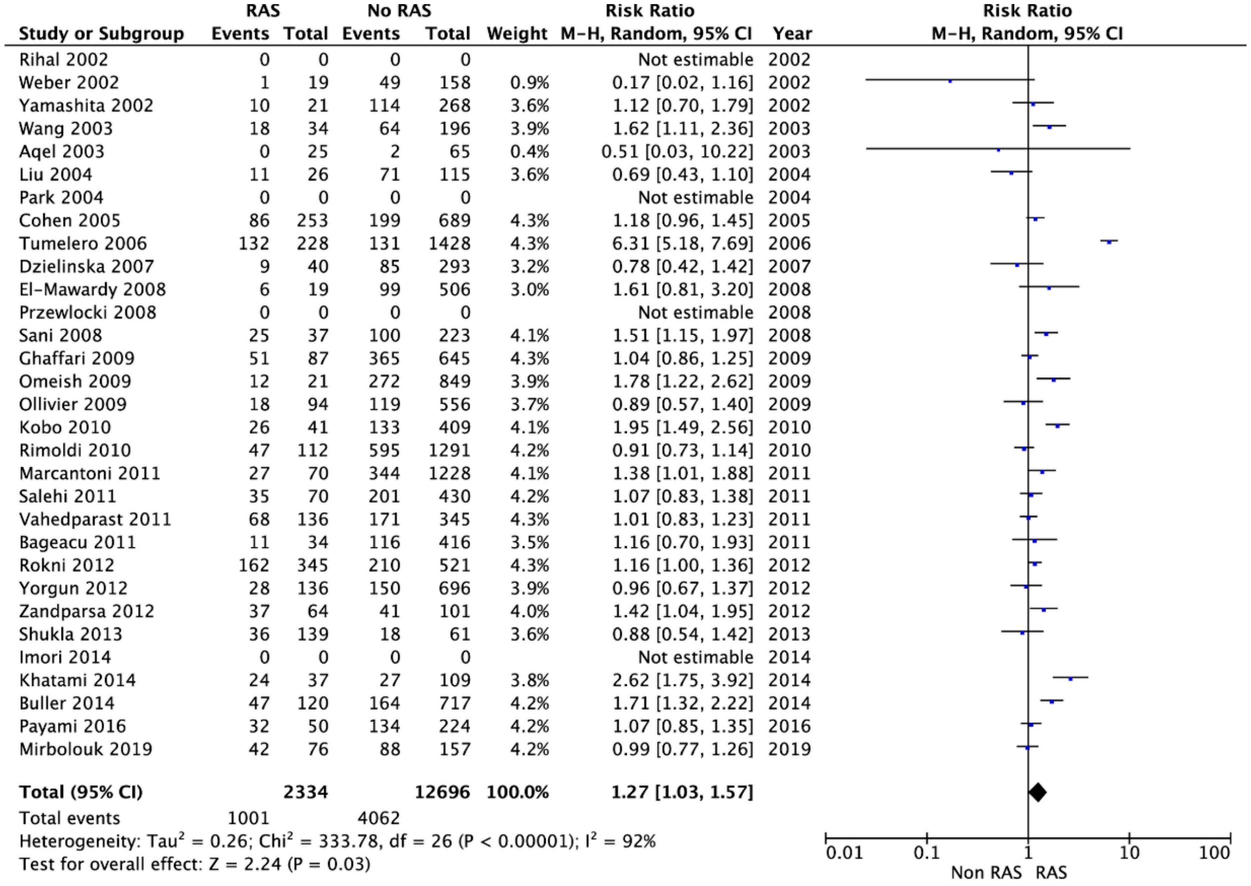

Supplemental Figure S7. Female sex as a risk factor for RAS

DIABETES MELLITUS

Study or Subgroup	RAS Events	RAS Total	No RAS Events	No RAS Total	Weight	Risk Ratio M-H, Random, 95% CI	Year
Weber 2002	13	19	47	158	3.7%	2.30 [1.56, 3.39]	2002
Yamashita 2002	5	21	45	268	1.4%	1.42 [0.63, 3.19]	2002
Rihal 2002	0	0	0	0		Not estimable	2002
Wang 2003	16	34	65	196	3.5%	1.42 [0.94, 2.13]	2003
Aqel 2003	7	25	35	65	1.9%	0.52 [0.27, 1.01]	2003
Liu 2004	6	26	15	115	1.3%	1.77 [0.76, 4.12]	2004
Park 2004	49	158	287	1301	5.0%	1.41 [1.09, 1.81]	2004
Cohen 2005	40	253	107	689	4.2%	1.02 [0.73, 1.42]	2005
Tumelero 2006	37	228	131	1428	4.1%	1.77 [1.26, 2.48]	2006
Dzielinska 2007	8	40	45	293	1.9%	1.30 [0.66, 2.56]	2007
El-Mawardy 2008	7	19	190	506	2.2%	0.98 [0.54, 1.79]	2008
Przewlocki 2008	0	0	0	0		Not estimable	2008
Sani 2008	17	37	71	223	3.6%	1.44 [0.97, 2.15]	2008
Ghaffari 2009	31	87	177	645	4.4%	1.30 [0.95, 1.77]	2009
Omeish 2009	11	21	307	849	3.4%	1.45 [0.95, 2.20]	2009
Ollivier 2009	26	94	132	556	3.9%	1.17 [0.81, 1.67]	2009
Kobo 2010	18	41	159	409	3.9%	1.13 [0.78, 1.63]	2010
Rimoldi 2010	38	112	225	1291	4.7%	1.95 [1.46, 2.59]	2010
Vahedparast 2011	38	136	108	345	4.4%	0.89 [0.65, 1.22]	2011
Bageacu 2011	8	34	74	416	2.0%	1.32 [0.70, 2.51]	2011
Marcantoni 2011	34	70	430	1228	5.0%	1.39 [1.08, 1.79]	2011
Salehi 2011	28	70	191	430	4.5%	0.90 [0.66, 1.22]	2011
Yorgun 2012	25	136	139	696	3.7%	0.92 [0.63, 1.35]	2012
Zandparsa 2012	25	64	46	101	3.8%	0.86 [0.59, 1.25]	2012
Rokni 2012	139	345	189	521	5.9%	1.11 [0.94, 1.32]	2012
Shukla 2013	20	139	8	61	1.6%	1.10 [0.51, 2.35]	2013
Buller 2014	39	120	225	717	4.7%	1.04 [0.78, 1.37]	2014
Imori 2014	0	0	0	0		Not estimable	2014
Khatami 2014	21	37	33	109	3.5%	1.87 [1.26, 2.80]	2014
Payami 2016	16	50	82	224	3.2%	0.87 [0.56, 1.36]	2016
Mirbolouk 2019	36	76	74	157	4.6%	1.00 [0.75, 1.34]	2019
**Total (95% CI)**		**2492**		**13997**	**100.0%**	**1.22 [1.10, 1.36]**	
Total events	758		3637				

Heterogeneity: $Tau^2 = 0.04$; $Chi^2 = 63.42$, df = 27 ($P < 0.0001$); $I^2 = 57\%$
Test for overall effect: Z = 3.61 (P = 0.0003)

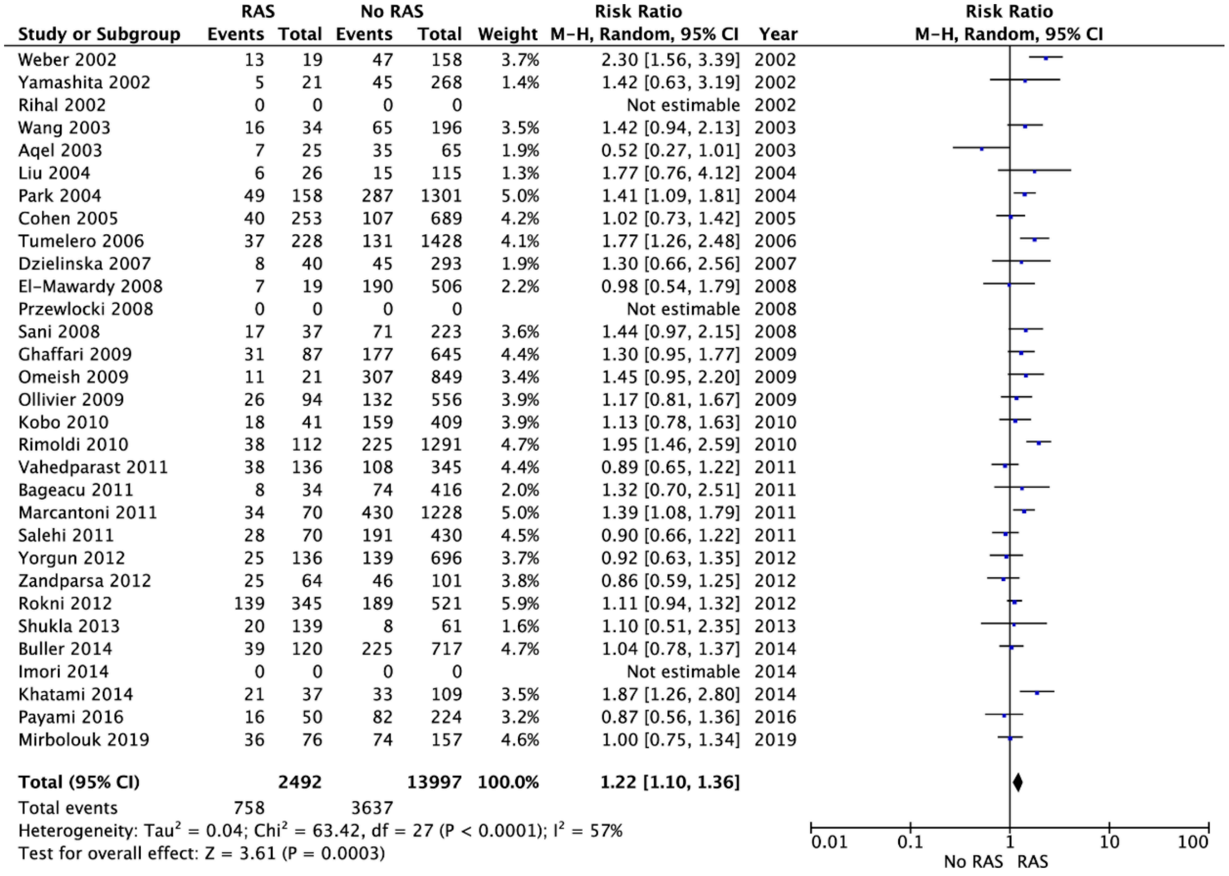

Supplemental Figure S8. Diabetes mellitus as a risk factor for RAS

ARTERIAL HYPERTENSION

Study or Subgroup	RAS Events	RAS Total	No RAS Events	No RAS Total	Weight	Risk Ratio M-H, Random, 95% CI	Year
Weber 2002	18	19	103	158	5.4%	1.45 [1.24, 1.70]	2002
Yamashita 2002	18	21	121	268	4.8%	1.90 [1.53, 2.36]	2002
Rihal 2002	0	0	0	0		Not estimable	2002
Wang 2003	29	34	124	196	5.2%	1.35 [1.13, 1.61]	2003
Aqel 2003	23	25	62	65	5.7%	0.96 [0.85, 1.10]	2003
Liu 2004	22	26	47	115	4.2%	2.07 [1.57, 2.72]	2004
Park 2004	104	158	575	1301	5.7%	1.49 [1.31, 1.69]	2004
Cohen 2005	202	253	428	689	6.1%	1.29 [1.18, 1.40]	2005
Tumelero 2006	0	0	0	0		Not estimable	2006
Dzielinska 2007	40	40	293	293	6.3%	1.00 [0.97, 1.03]	2007
Sani 2008	32	37	143	223	5.4%	1.35 [1.15, 1.58]	2008
El-Mawardy 2008	0	0	0	0		Not estimable	2008
Przewlocki 2008	0	0	0	0		Not estimable	2008
Ghaffari 2009	0	0	0	0		Not estimable	2009
Omeish 2009	13	21	215	849	3.4%	2.44 [1.71, 3.49]	2009
Ollivier 2009	78	94	345	556	5.8%	1.34 [1.20, 1.50]	2009
Kobo 2010	41	41	409	409		Not estimable	2010
Rimoldi 2010	112	112	1291	1291		Not estimable	2010
Vahedparast 2011	136	136	345	345		Not estimable	2011
Bageacu 2011	20	34	176	416	3.9%	1.39 [1.03, 1.88]	2011
Marcantoni 2011	69	70	1068	1228	6.3%	1.13 [1.09, 1.17]	2011
Salehi 2011	58	70	315	430	5.8%	1.13 [1.00, 1.28]	2011
Yorgun 2012	136	136	696	696		Not estimable	2012
Zandparsa 2012	55	64	64	101	5.2%	1.36 [1.13, 1.62]	2012
Rokni 2012	294	345	393	521	6.2%	1.13 [1.06, 1.21]	2012
Shukla 2013	106	139	29	61	4.1%	1.60 [1.21, 2.12]	2013
Buller 2014	0	0	0	0		Not estimable	2014
Imori 2014	0	0	0	0		Not estimable	2014
Khatami 2014	32	37	68	109	5.0%	1.39 [1.14, 1.68]	2014
Payami 2016	50	50	224	224		Not estimable	2016
Mirbolouk 2019	58	76	121	157	5.5%	0.99 [0.85, 1.15]	2019
**Total (95% CI)**		**1563**		**7736**	**100.0%**	**1.33 [1.21, 1.46]**	
Total events	1271		4690				

Heterogeneity: $Tau^2 = 0.04$; $Chi^2 = 305.45$, df = 18 (P < 0.00001); $I^2 = 94\%$
Test for overall effect: Z = 5.79 (P < 0.00001)

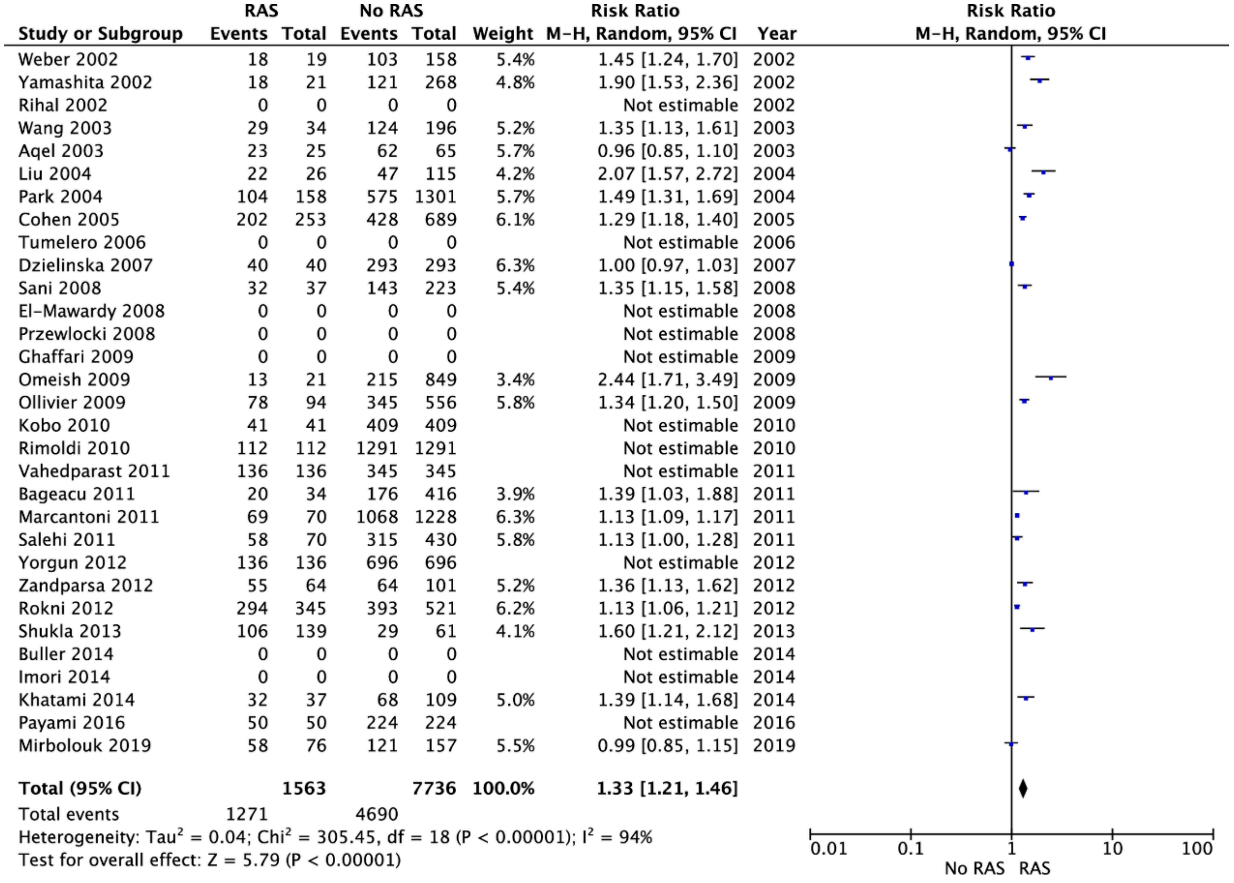

Supplemental Figure S9. Arterial hypertension as a risk factor for RAS

DYSLIPIDEMIA

Study or Subgroup	RAS Events	RAS Total	No RAS Events	No RAS Total	Weight	Risk Ratio M–H, Fixed, 95% CI	Year
Rihal 2002	0	0	0	0		Not estimable	2002
Weber 2002	15	19	96	158	1.1%	1.30 [1.00, 1.69]	2002
Yamashita 2002	8	21	83	268	0.7%	1.23 [0.69, 2.18]	2002
Wang 2003	17	34	97	196	1.6%	1.01 [0.70, 1.45]	2003
Aqel 2003	19	25	49	65	1.5%	1.01 [0.78, 1.31]	2003
Liu 2004	15	26	38	115	0.8%	1.75 [1.15, 2.66]	2004
Park 2004	29	152	135	1227	1.6%	1.73 [1.20, 2.50]	2004
Cohen 2005	169	253	441	689	13.0%	1.04 [0.94, 1.16]	2005
Tumelero 2006	0	0	0	0		Not estimable	2006
Dzielinska 2007	35	40	216	293	2.8%	1.19 [1.04, 1.36]	2007
El–Mawardy 2008	0	0	0	0		Not estimable	2008
Przewlocki 2008	0	0	0	0		Not estimable	2008
Sani 2008	28	37	137	223	2.1%	1.23 [1.00, 1.52]	2008
Ghaffari 2009	45	87	329	645	4.3%	1.01 [0.82, 1.26]	2009
Omeish 2009	0	0	0	0		Not estimable	2009
Ollivier 2009	70	94	416	556	6.6%	1.00 [0.88, 1.13]	2009
Kobo 2010	27	41	191	409	1.9%	1.41 [1.11, 1.80]	2010
Rimoldi 2010	106	112	1037	1291	9.1%	1.18 [1.12, 1.24]	2010
Marcantoni 2011	64	70	909	1228	5.4%	1.24 [1.14, 1.34]	2011
Salehi 2011	65	70	380	430	5.8%	1.05 [0.98, 1.13]	2011
Vahedparast 2011	53	136	154	345	4.8%	0.87 [0.69, 1.11]	2011
Bageacu 2011	11	34	164	416	1.4%	0.82 [0.50, 1.35]	2011
Rokni 2012	276	345	359	521	15.7%	1.16 [1.07, 1.26]	2012
Yorgun 2012	87	136	447	696	8.0%	1.00 [0.87, 1.14]	2012
Zandparsa 2012	49	64	80	101	3.4%	0.97 [0.82, 1.14]	2012
Shukla 2013	35	139	18	61	1.4%	0.85 [0.53, 1.38]	2013
Imori 2014	0	0	0	0		Not estimable	2014
Khatami 2014	25	37	56	109	1.6%	1.32 [0.99, 1.75]	2014
Buller 2014	0	0	0	0		Not estimable	2014
Payami 2016	20	50	106	224	2.1%	0.85 [0.59, 1.22]	2016
Mirbolouk 2019	53	76	97	157	3.5%	1.13 [0.93, 1.37]	2019
**Total (95% CI)**		**2098**		**10423**	**100.0%**	**1.10 [1.06, 1.14]**	
Total events	1321		6035				

Heterogeneity: $Chi^2 = 55.75$, df = 23 (P = 0.0002); $I^2 = 59\%$

Test for overall effect: Z = 5.35 (P < 0.00001)

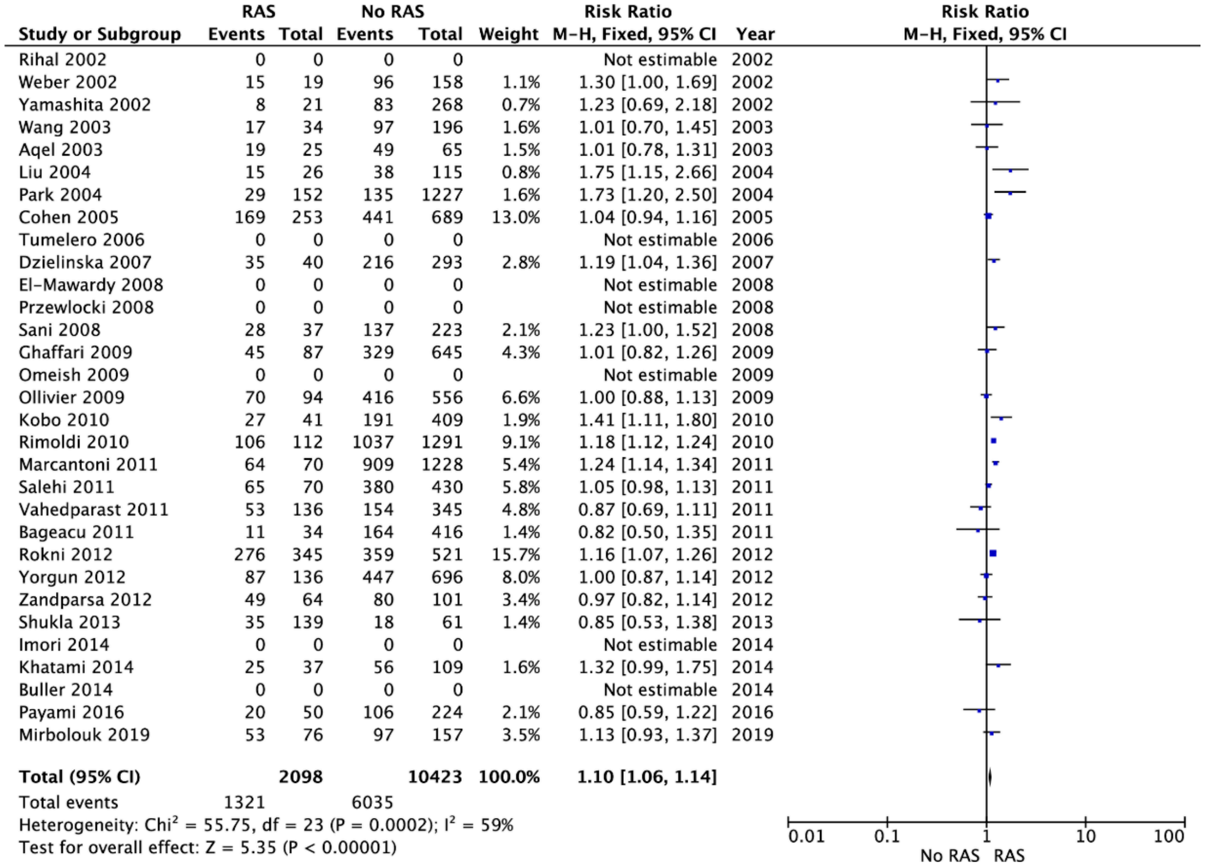

Supplemental Figure S10. Dyslipidemia as a risk factor for RAS

PERIPHERAL ARTERY DISEASE

Study or Subgroup	RAS Events	RAS Total	No RAS Events	No RAS Total	Weight	Risk Ratio M-H, Random, 95% CI	Year
Rihal 2002	11	57	26	240	7.3%	1.78 [0.94, 3.39]	2002
Weber 2002	0	0	0	0		Not estimable	2002
Yamashita 2002	0	0	0	0		Not estimable	2002
Wang 2003	6	34	21	196	6.5%	1.65 [0.72, 3.78]	2003
Aqel 2003	14	25	18	65	7.8%	2.02 [1.20, 3.42]	2003
Liu 2004	0	0	0	0		Not estimable	2004
Park 2004	43	158	103	1301	8.5%	3.44 [2.51, 4.71]	2004
Cohen 2005	57	253	64	689	8.5%	2.43 [1.75, 3.36]	2005
Tumelero 2006	0	0	0	0		Not estimable	2006
Dzielinska 2007	39	40	223	293	9.0%	1.28 [1.18, 1.39]	2007
El-Mawardy 2008	0	0	0	0		Not estimable	2008
Przewlocki 2008	0	0	0	0		Not estimable	2008
Sani 2008	0	0	0	0		Not estimable	2008
Ollivier 2009	22	94	103	556	8.3%	1.26 [0.84, 1.89]	2009
Ghaffari 2009	6	87	15	645	6.2%	2.97 [1.18, 7.44]	2009
Omeish 2009	0	0	0	0		Not estimable	2009
Kobo 2010	0	0	0	0		Not estimable	2010
Rimoldi 2010	41	112	88	1291	8.5%	5.37 [3.91, 7.37]	2010
Marcantoni 2011	32	70	209	1228	8.6%	2.69 [2.02, 3.57]	2011
Salehi 2011	0	0	0	0		Not estimable	2011
Vahedparast 2011	0	0	0	0		Not estimable	2011
Bageacu 2011	0	0	0	0		Not estimable	2011
Rokni 2012	28	345	29	521	7.9%	1.46 [0.88, 2.41]	2012
Yorgun 2012	7	136	25	696	6.6%	1.43 [0.63, 3.25]	2012
Zandparsa 2012	0	0	0	0		Not estimable	2012
Shukla 2013	23	139	5	61	6.2%	2.02 [0.81, 5.06]	2013
Imori 2014	0	0	0	0		Not estimable	2014
Khatami 2014	0	0	0	0		Not estimable	2014
Buller 2014	0	0	0	0		Not estimable	2014
Payami 2016	0	0	0	0		Not estimable	2016
Mirbolouk 2019	0	0	0	0		Not estimable	2019
**Total (95% CI)**		**1550**		**7782**	**100.0%**	**2.11 [1.40, 3.16]**	
Total events	329		929				

Heterogeneity: $Tau^2 = 0.48$; $Chi^2 = 206.07$, df = 12 ($P < 0.00001$); $I^2 = 94\%$

Test for overall effect: Z = 3.59 (P = 0.0003)

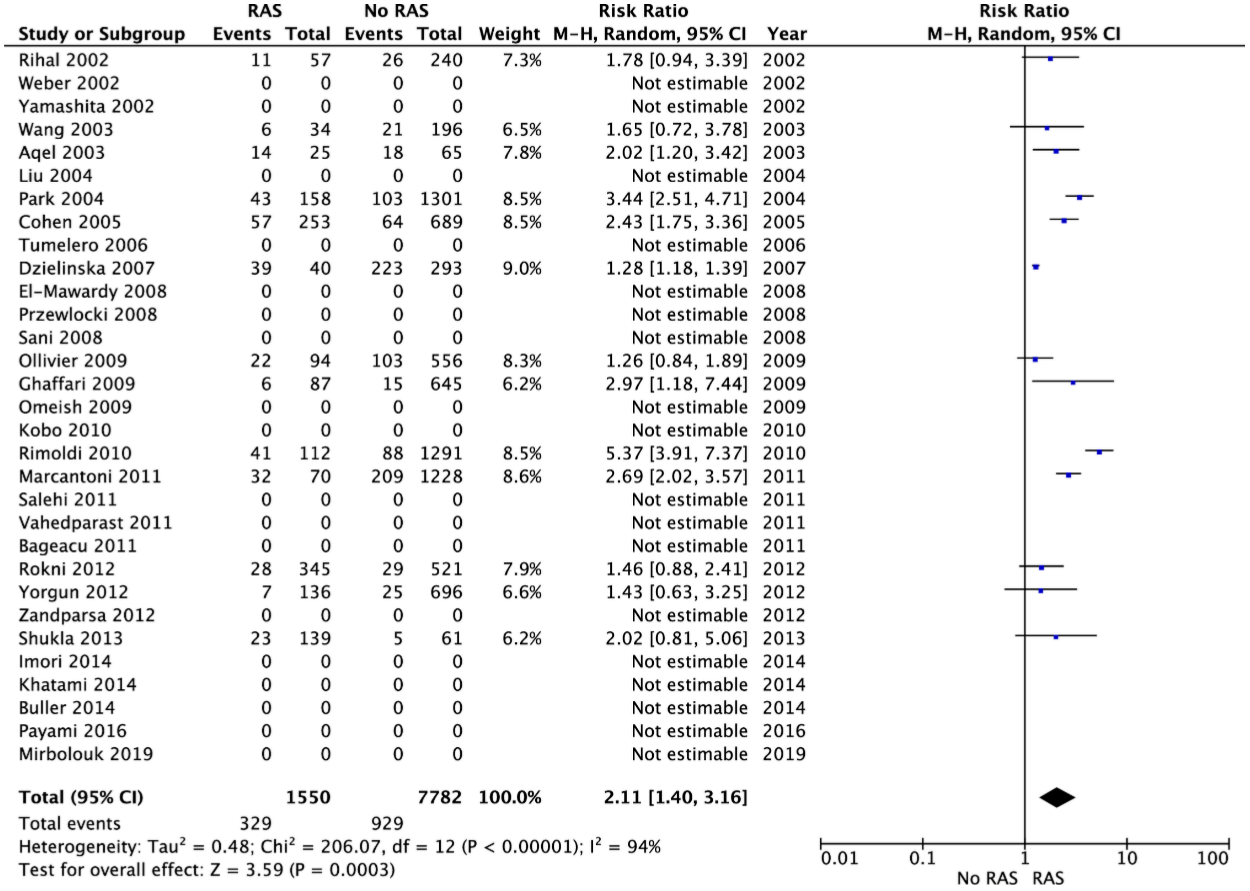

Supplemental Figure S11. Peripheral artery disease as a risk factor for RAS

SMOKING

Study or Subgroup	RAS Events	RAS Total	No RAS Events	No RAS Total	Weight	Risk Ratio M-H, Fixed, 95% CI	Year
Rihal 2002	0	0	0	0		Not estimable	2002
Weber 2002	5	19	28	158	0.5%	1.48 [0.65, 3.39]	2002
Yamashita 2002	8	21	83	268	1.0%	1.23 [0.69, 2.18]	2002
Aqel 2003	12	25	22	65	1.1%	1.42 [0.83, 2.41]	2003
Wang 2003	17	34	102	196	2.6%	0.96 [0.67, 1.38]	2003
Liu 2004	0	0	0	0		Not estimable	2004
Park 2004	88	158	676	1301	12.7%	1.07 [0.92, 1.24]	2004
Cohen 2005	57	253	146	689	6.8%	1.06 [0.81, 1.39]	2005
Tumelero 2006	0	0	0	0		Not estimable	2006
Dzielinska 2007	26	40	188	293	3.9%	1.01 [0.79, 1.29]	2007
El-Mawardy 2008	10	19	262	506	1.6%	1.02 [0.66, 1.57]	2008
Przewlocki 2008	0	0	0	0		Not estimable	2008
Sani 2008	3	37	36	223	0.9%	0.50 [0.16, 1.55]	2008
Ollivier 2009	48	94	315	556	7.9%	0.90 [0.73, 1.11]	2009
Ghaffari 2009	13	87	132	645	2.7%	0.73 [0.43, 1.23]	2009
Omeish 2009	9	21	277	849	1.2%	1.31 [0.79, 2.17]	2009
Kobo 2010	0	0	0	0		Not estimable	2010
Rimoldi 2010	28	112	231	1291	3.2%	1.40 [0.99, 1.97]	2010
Bageacu 2011	17	34	170	416	2.2%	1.22 [0.86, 1.75]	2011
Marcantoni 2011	39	70	786	1228	7.4%	0.87 [0.70, 1.08]	2011
Salehi 2011	13	70	95	430	2.3%	0.84 [0.50, 1.42]	2011
Vahedparast 2011	50	136	151	345	7.4%	0.84 [0.65, 1.08]	2011
Rokni 2012	58	345	120	521	8.3%	0.73 [0.55, 0.97]	2012
Yorgun 2012	74	136	368	696	10.5%	1.03 [0.87, 1.22]	2012
Zandparsa 2012	29	64	34	101	2.3%	1.35 [0.92, 1.98]	2012
Shukla 2013	20	139	7	61	0.8%	1.25 [0.56, 2.81]	2013
Buller 2014	73	120	403	717	10.0%	1.08 [0.92, 1.27]	2014
Imori 2014	0	0	0	0		Not estimable	2014
Khatami 2014	7	37	36	109	1.6%	0.57 [0.28, 1.18]	2014
Payami 2016	10	50	27	224	0.9%	1.66 [0.86, 3.20]	2016
Mirbolouk 2019	0	0	0	0		Not estimable	2019
**Total (95% CI)**		**2121**		**11888**	**100.0%**	**1.00 [0.94, 1.06]**	
Total events	714		4695				

Heterogeneity: $Chi^2 = 31.02$, df = 23 (P = 0.12); $I^2 = 26\%$

Test for overall effect: Z = 0.09 (P = 0.93)

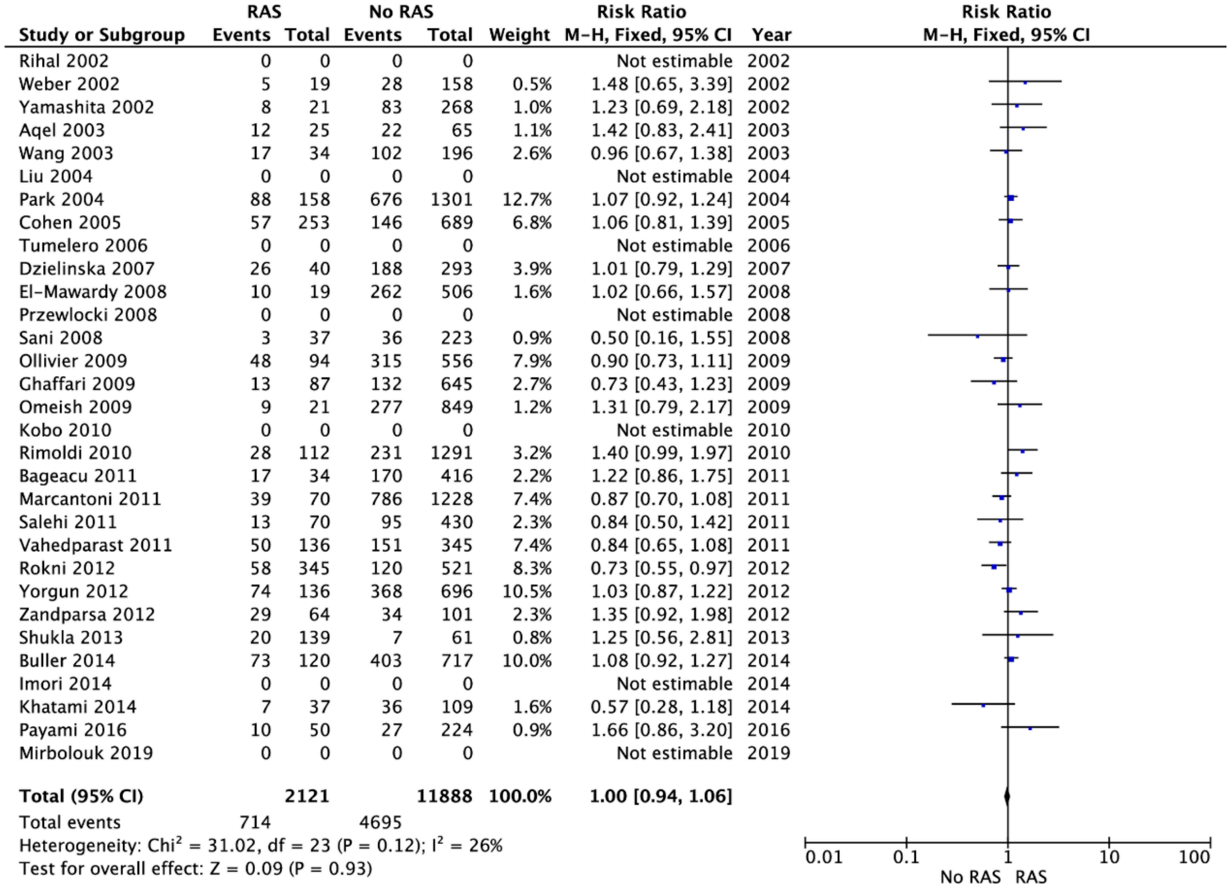

Supplemental Figure S12. Smoking as a risk factor for RAS

CHRONIC KIDNEY DISEASE

Study or Subgroup	RAS Events	RAS Total	No RAS Events	No RAS Total	Weight	Risk Ratio M-H, Random, 95% CI	Year
Weber 2002	1	19	7	158	1.4%	1.19 [0.15, 9.14]	2002
Yamashita 2002	0	0	0	0		Not estimable	2002
Rihal 2002	0	0	0	0		Not estimable	2002
Wang 2003	0	0	0	0		Not estimable	2003
Aqel 2003	0	0	0	0		Not estimable	2003
Liu 2004	8	26	6	115	4.5%	5.90 [2.24, 15.54]	2004
Park 2004	23	158	53	785	9.4%	2.16 [1.36, 3.41]	2004
Cohen 2005	46	253	14	689	7.9%	8.95 [5.01, 15.99]	2005
Tumelero 2006	0	0	0	0		Not estimable	2006
Dzielinska 2007	13	40	39	293	8.4%	2.44 [1.43, 4.16]	2007
Sani 2008	0	0	0	0		Not estimable	2008
El-Mawardy 2008	0	0	0	0		Not estimable	2008
Przewlocki 2008	0	0	0	0		Not estimable	2008
Ghaffari 2009	0	0	0	0		Not estimable	2009
Omeish 2009	0	0	0	0		Not estimable	2009
Ollivier 2009	30	94	71	556	10.6%	2.50 [1.73, 3.61]	2009
Kobo 2010	9	41	41	409	7.2%	2.19 [1.15, 4.18]	2010
Rimoldi 2010	53	112	198	1291	12.2%	3.09 [2.44, 3.90]	2010
Vahedparast 2011	0	0	0	0		Not estimable	2011
Bageacu 2011	0	0	0	0		Not estimable	2011
Marcantoni 2011	14	70	74	1228	8.6%	3.32 [1.98, 5.57]	2011
Salehi 2011	0	0	0	0		Not estimable	2011
Yorgun 2012	0	0	0	0		Not estimable	2012
Zandparsa 2012	0	0	0	0		Not estimable	2012
Rokni 2012	74	345	63	521	11.3%	1.77 [1.30, 2.41]	2012
Shukla 2013	31	139	9	61	6.8%	1.51 [0.77, 2.98]	2013
Buller 2014	0	0	0	0		Not estimable	2014
Imori 2014	0	0	0	0		Not estimable	2014
Khatami 2014	0	0	0	0		Not estimable	2014
Payami 2016	5	50	18	224	4.7%	1.24 [0.49, 3.19]	2016
Mirbolouk 2019	17	76	14	157	7.1%	2.51 [1.31, 4.82]	2019
**Total (95% CI)**		**1423**		**6487**	**100.0%**	**2.62 [2.04, 3.37]**	
Total events	324		607				

Heterogeneity: $Tau^2 = 0.12$; $Chi^2 = 35.47$, df = 12 (P = 0.0004); $I^2 = 66\%$

Test for overall effect: Z = 7.55 (P < 0.00001)

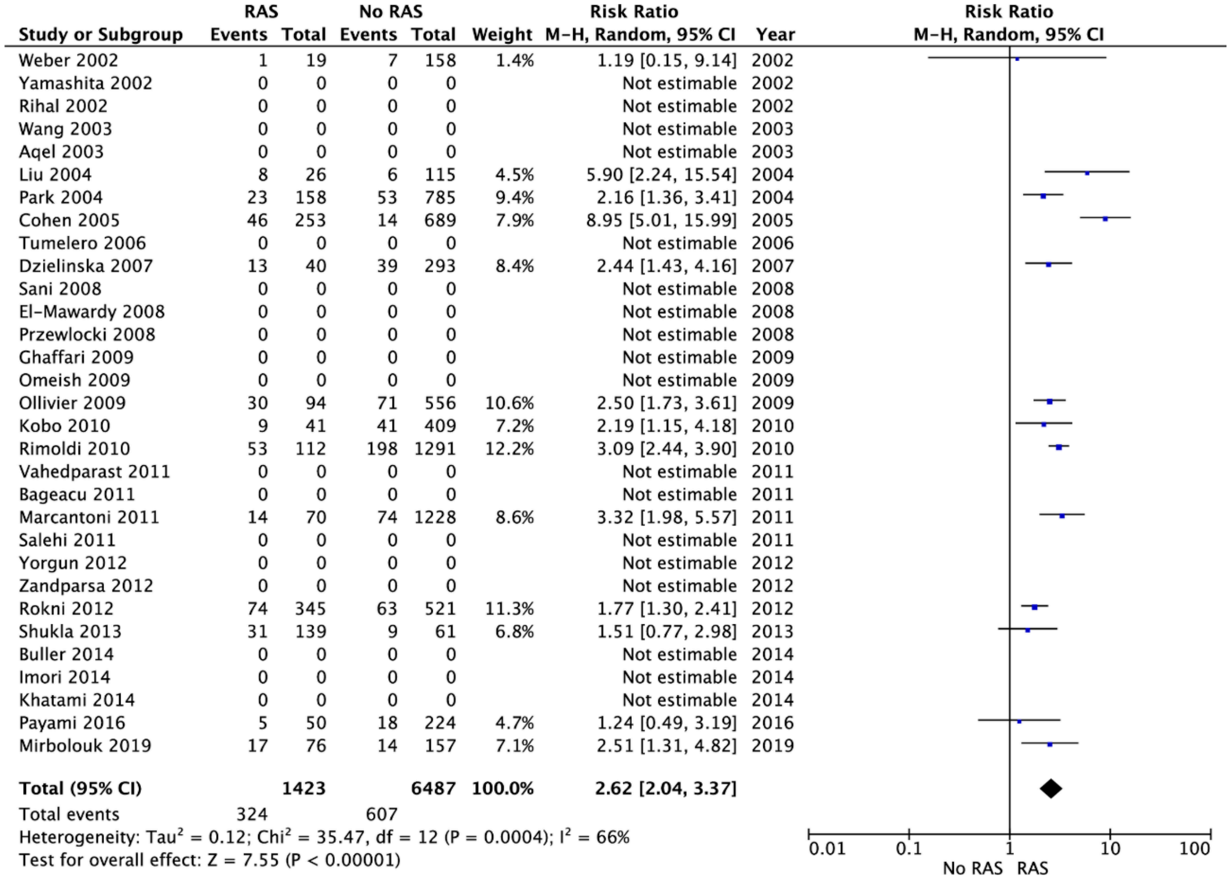

Supplemental Figure S13. Chronic kidney disease as a risk factor for RAS

THREE-VESSEL CORONARY ARTERY DISEASE

Study or Subgroup	RAS Events	RAS Total	No RAS Events	No RAS Total	Weight	Risk Ratio M-H, Random, 95% CI	Year
Rihal 2002	0	0	0	0		Not estimable	2002
Weber 2002	0	0	0	0		Not estimable	2002
Yamashita 2002	0	0	0	0		Not estimable	2002
Aqel 2003	17	25	37	65	6.4%	1.19 [0.85, 1.68]	2003
Wang 2003	14	34	52	196	5.4%	1.55 [0.98, 2.47]	2003
Liu 2004	11	26	0	115	0.4%	98.81 [6.01, 1625.43]	2004
Park 2004	66	158	256	1301	7.5%	2.12 [1.71, 2.63]	2004
Cohen 2005	0	0	0	0		Not estimable	2005
Tumelero 2006	0	0	0	0		Not estimable	2006
Dzielinska 2007	17	40	77	293	5.9%	1.62 [1.08, 2.43]	2007
El-Mawardy 2008	0	0	0	0		Not estimable	2008
Przewlocki 2008	0	0	0	0		Not estimable	2008
Sani 2008	0	0	0	0		Not estimable	2008
Ollivier 2009	0	0	0	0		Not estimable	2009
Ghaffari 2009	31	87	189	645	6.7%	1.22 [0.89, 1.65]	2009
Omeish 2009	2	21	43	849	1.5%	1.88 [0.49, 7.25]	2009
Kobo 2010	0	0	0	0		Not estimable	2010
Rimoldi 2010	0	0	0	0		Not estimable	2010
Bageacu 2011	12	34	93	416	5.2%	1.58 [0.97, 2.57]	2011
Marcantoni 2011	20	70	209	1228	6.0%	1.68 [1.14, 2.48]	2011
Salehi 2011	40	70	127	430	7.2%	1.93 [1.51, 2.48]	2011
Vahedparast 2011	0	0	0	0		Not estimable	2011
Rokni 2012	185	345	182	521	7.9%	1.54 [1.32, 1.79]	2012
Yorgun 2012	26	136	59	696	5.7%	2.26 [1.48, 3.44]	2012
Zandparsa 2012	31	64	51	101	6.6%	0.96 [0.70, 1.32]	2012
Shukla 2013	70	139	20	61	6.0%	1.54 [1.03, 2.28]	2013
Buller 2014	53	120	333	717	7.5%	0.95 [0.77, 1.18]	2014
Imori 2014	37	116	169	1606	6.8%	3.03 [2.24, 4.10]	2014
Khatami 2014	0	0	0	0		Not estimable	2014
Payami 2016	0	0	0	0		Not estimable	2016
Mirbolouk 2019	45	76	86	157	7.3%	1.08 [0.85, 1.37]	2019
**Total (95% CI)**		**1561**		**9397**	**100.0%**	**1.56 [1.30, 1.87]**	
Total events	677		1983				

Heterogeneity: $Tau^2 = 0.10$; $Chi^2 = 83.64$, df = 16 ($P < 0.00001$); $I^2 = 81\%$
Test for overall effect: Z = 4.81 ($P < 0.00001$)

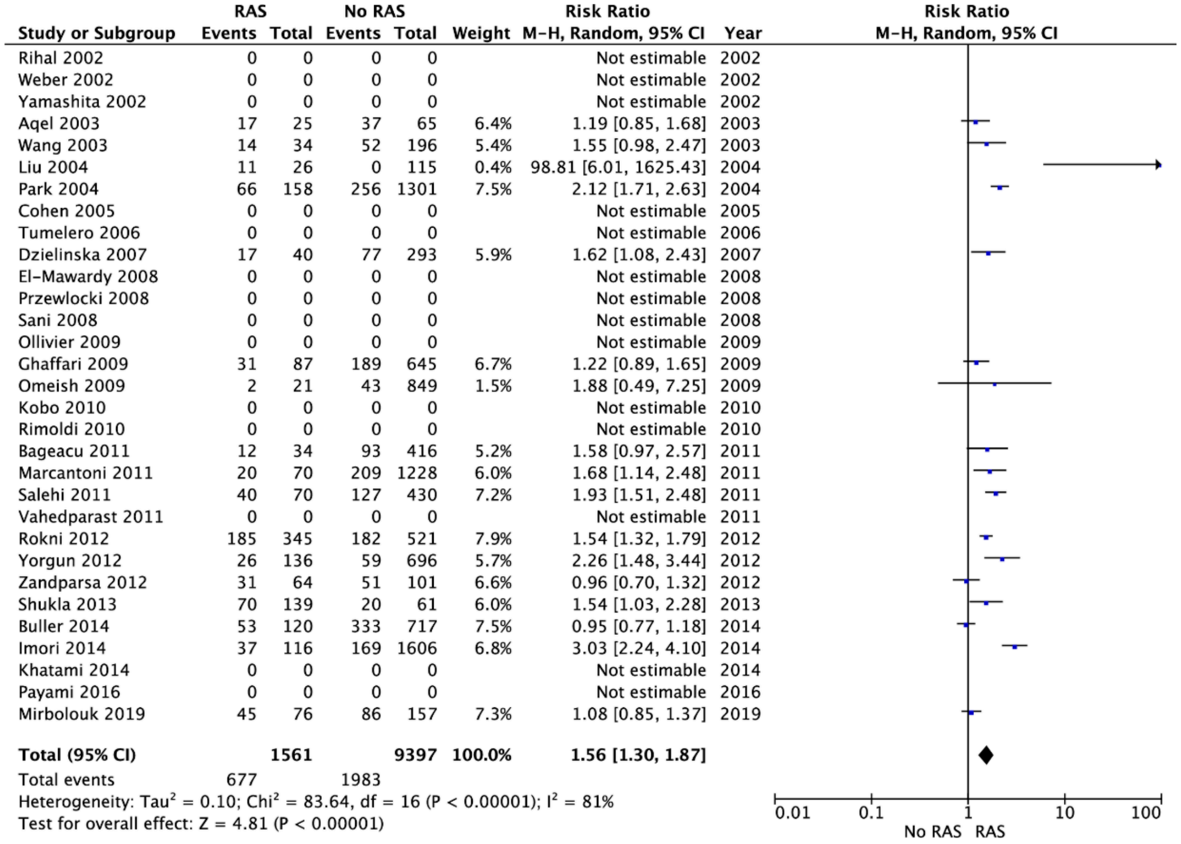

Supplemental Figure S14. Three-vessel coronary artery disease as a risk factor for RAS

LEFT CORONARY ARTERY MAIN STEM DISEASE

Study or Subgroup	RAS Events	RAS Total	No RAS Events	No RAS Total	Weight	Risk Ratio M-H, Random, 95% CI	Year
Rihal 2002	0	0	0	0		Not estimable	2002
Weber 2002	0	0	0	0		Not estimable	2002
Yamashita 2002	0	0	0	0		Not estimable	2002
Aqel 2003	0	0	0	0		Not estimable	2003
Wang 2003	0	0	0	0		Not estimable	2003
Liu 2004	0	0	0	0		Not estimable	2004
Park 2004	18	158	110	1301	15.3%	1.35 [0.84, 2.16]	2004
Cohen 2005	0	0	0	0		Not estimable	2005
Tumelero 2006	0	0	0	0		Not estimable	2006
Dzielinska 2007	0	0	0	0		Not estimable	2007
El-Mawardy 2008	0	0	0	0		Not estimable	2008
Przewlocki 2008	0	0	0	0		Not estimable	2008
Sani 2008	1	37	4	223	2.1%	1.51 [0.17, 13.11]	2008
Ghaffari 2009	0	0	0	0		Not estimable	2009
Omeish 2009	2	21	8	849	4.0%	10.11 [2.28, 44.75]	2009
Ollivier 2009	0	0	0	0		Not estimable	2009
Kobo 2010	0	0	0	0		Not estimable	2010
Rimoldi 2010	0	0	0	0		Not estimable	2010
Bageacu 2011	0	0	0	0		Not estimable	2011
Marcantoni 2011	9	70	86	1228	12.0%	1.84 [0.97, 3.49]	2011
Salehi 2011	6	70	20	430	8.6%	1.84 [0.77, 4.43]	2011
Vahedparast 2011	0	0	0	0		Not estimable	2011
Rokni 2012	20	345	18	521	12.4%	1.68 [0.90, 3.13]	2012
Yorgun 2012	5	136	3	696	4.3%	8.53 [2.06, 35.27]	2012
Zandparsa 2012	0	0	0	0		Not estimable	2012
Shukla 2013	20	139	7	61	9.5%	1.25 [0.56, 2.81]	2013
Buller 2014	18	120	108	717	15.5%	1.00 [0.63, 1.58]	2014
Imori 2014	21	116	134	1606	16.4%	2.17 [1.43, 3.30]	2014
Khatami 2014	0	0	0	0		Not estimable	2014
Payami 2016	0	0	0	0		Not estimable	2016
Mirbolouk 2019	0	0	0	0		Not estimable	2019
**Total (95% CI)**		**1212**		**7632**	**100.0%**	**1.78 [1.28, 2.47]**	
Total events	120		498				

Heterogeneity: $Tau^2 = 0.12$; $Chi^2 = 18.62$, df = 9 (P = 0.03); $I^2 = 52\%$
Test for overall effect: Z = 3.44 (P = 0.0006)

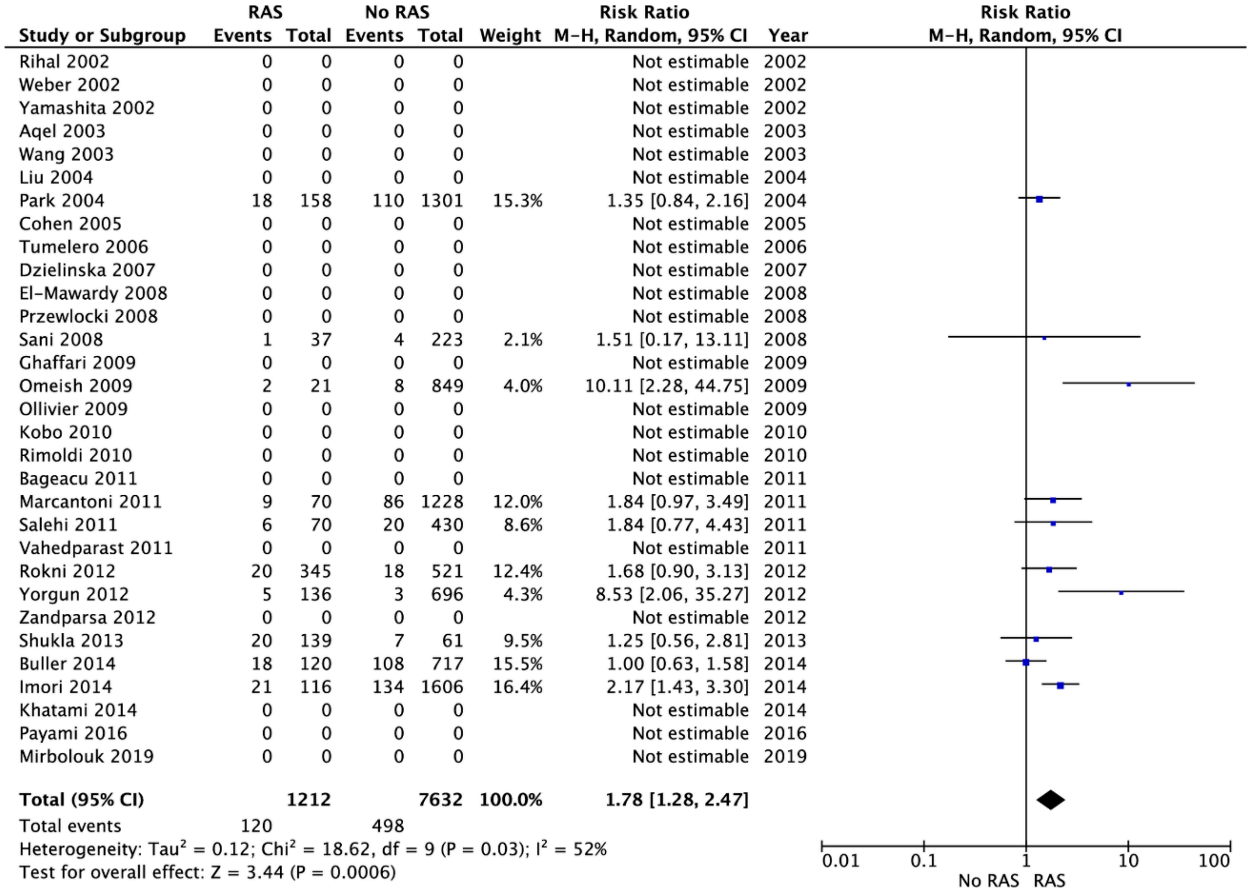

Supplemental Figure S15. Left coronary artery main stem disease as a risk factor for RAS

SUPPLEMENTAL TABLES

Supplemental Table S1. Baseline demographic, comorbidity, and clinical characteristics of patients across studies included in the analysis

Authors of the study and publication year	Average age (years)	Female sex N/N total (%)	Diabetes mellitus N/N total (%)	Arterial hypertension N/N total (%)	Dyslipidemia N/N total (%)	Smoking N/N total (%)	Renal failure N/N total (%)	PVD N/N total (%)	CAS N/N total (%)	Previous MI N/N total (%)
**Rihal et al. 2002**	64.9±10.2	123/297 (58.2)	67/297 (22.6)	297/297 (100)	124/297 (47.1)	172/297 (57.9)	-	-	-	80/297 (26.9)
**Weber et al. 2002**	61±10	62/177 (35.0)	60/177 (33.9)	159/177 (89.8)	-	33/177 (18.6)	-	-	-	46/177 (26.0)
**Yamashita et al. 2002**	65.8±10.6	125/289 (43.3)	81/289 (28.0)	138/289 (47.8)	91/289 (31.5)	90/289 (31.1)	-	-	-	-
**Aqel et al. 2003**	65.3±9.4	2/90 (2.2)	42/90 (46.7)	85/90 (94.4)	68/90 (75.6)	34/90 (37.0)	-	-	-	15/90 (16.7)
**Wang et al. 2003**	65.1±10.2	82/230 (35.7)	81/230 (35.2)	153/230 (66.5)	-	119/230 (51.8)	-	27/230 (11.7)	44/230 (19.1)	52/230 (22.7)
**Liu et al. 2004**	59±10	82/141 (58.2)	21/14 (14.9)	69/141 (48.9)	17/141 (12.1)	-	14/141 (9.9)	-	-	-
**Park et al. 2004**	59.2±9.9	Male:female 2.05:1 (N=1301)	287/1301 (22.1)	575/1301 (44.2)	-	676/1299 (52.0)	53/785 (6.8)	103/1301 (7.9)	46/1186 (3.9)	-
**Cohen et al. 2005**	64 (55-73)	252/843 (29.9)	133/843 (15.8)	549/843 (65.1)	-	181/843 (21.1)	40/843 (4.7)	-	-	141/843 (16.7)
**Dzielinska et al. 2006**	56.6±9.5 without RAS 59.8±9.6 with RAS	94/333 (28.2)	53/333 (15.9)	333/333 (100)	251/333 (75.3)	214/333 (64.2)	53/333 (15.9)	262/333 (78.6)	261/333 (78.3)	-
**Tumelero et al. 2006**	61.6±11.8	765/1656 (46.2)	169/1656 (10.2)	1199/1656 (72.4)	-	-	-	-	-	-
**Ollivier et al. 2008**	67±10	137/650 (20.0)	158/650 (24.3)	423/650 (65.0)	486/650 (74.7)	383/650 (58.9)	101/650 (15.5)	125/650 (19.2)	-	161/650 (24.7)
**El-Mawardy et al. 2008**	52.6±8.5	122/525 (23.2)	197/525 (37.5)	525/525 (100)	-	272/525 (51.8)	-	-	-	127/525 (24.0)

**Przewlocki et al. 2008**	62.1±9.7	336/1036 (32.4)	189/1036 (18.2)	832/1036 (80.3)	899/1036 (86.8)	566/1036 (54.6)	-	-	80/1036 (7.7)	312/1036 (30.1)
**Sani et al. 2008**	57.2±10.1	125/260 (48.1)	88/260 (33.8)	175/260 (67.3)	165/260 (63.5)	39/260 (15.0)	-	-	-	-
**Ghaffari et al. 2009**	59.0±9.7	416/732 (56.8)	208/732 (28.4)	732/732 (100)	374/732 (51.1)	145/732 (19.8)		21/732 (2.9)		114/732 (15.6)
**Omeish et al. 2009**	64.5±9.5	284/870 (32.6)	318/870 (36.6)	228/870 (26.2)	-	286/870 (32.9)	-	-	-	-
**Kobo et al. 2010**	65.7±9.8	130/450 (28.9)	122/450 (27.1)	300/450 (66.7)	160/450 (35.6)	-	43/450 (9.6)	-	-	-
**Rimoldi et al. 2010**	67±9.5	642/1403 (45.8)	263/1403 (16.8)	1403/1403 (100)	1143/1403 (81.5)	259/1403 (18.5)	251/1403 (17.9)	129/1403 (9.2)	-	-
**Bageacu et al. 2011**	62.1±10.8	127/450 (28.2)	82/450 (18.2)	196/450 (43.6)	-	187/450 (41.6)	-	-	-	-
**Marcantoni et al. 2011**	64±10	371/1298 (28.6)	467/1298 (36.0)	1129/1298 (87.0)	974/1298 (75.0)	831/1298 (64.0)	-	247/1298 (19.0)	-	351/1298 (27.0)
**Salehi et al. 2011**	60.1±9.4	236/500 (47.2)	219/500 (43.8)	373/500 (74.6)	445/500 (89.0)	108/500 (21.6)	-	-	-	-
**Vahedparast et al. 2011**	59.3±10.8	239/481 (49.7)	146/481 (30.4)	481/481 (100)	207/481 (43.0)	201/481 (41.8)	-	-	-	-
**Rokni et al. 2012**	63.1±10.3	372/866 (43.0)	328/866 (37.9)	687/866 (79.3)	635/866 (73.3)	178/866 (20.6)	137/866 (15.8)	57/866 (6.6)	-	309/866 (35.7)
**Yorgun et al. 2012**	61.2±8.2	178/832 (21.4)	164/832 (12.7)	832/832 (100)	534/832 (64.2)	442/832 (53.1)	-	32/832 (3.8)	-	-
**Zandparsa et al. 2012**	59.9±8.9	78/165 (47.3)	71/165 (43)	119/165 (72.1)	129/165 (78.2)	63/165 (38.2)	-	-	-	-
**Shukla et al. 2013**	-	-	-	-	-	-	-	-	-	-
**Buller et al. 2014**	67.0±9.9	211/837 (25.2)	264/837 (31.5)	264/837 (31.5)	-	476/837 (56.9)	-	-	-	-
**Imori et al. 2014**	71±9	527/1734 (30.4)	524/1734 (30.2)	1179/1734 (68.0)	1104/1734 (63.6)	819/1734 (47.2)	-	-	-	234/1734 (18.7)
**Khatami et al. 2014**	61.2±9.5	51/146 (34.9)	54/146 (37.0)	100/146 (68.5)	81/146 (55.5)	43/146 (29.5)	-	-	-	-
**Payami et al. 2016**	60.8±10.9	166/274 (60.6)	98/274 (35.8)	274/274 (100)	126/274 (46.0)	37/274 (13.5)	23/274 (8.4)	-	-	-
**Mirbolouk et al. 2019**	64±10	130/233 (55.8)	110/233 (47.2)	179/233 (76.8)	-	-	-	-	-	46/233 (19.7)

Abbreviations: CAS-carotid artery stenosis; **MI-**myocardial infarction; **PVD-**peripheral vascular disease

Supplemental Table S2. Angiographic characteristics of renal artery stenosis (RAS) and coronary artery disease (CAD) across included studies

**Authors of the study and publication year**	**RAS ≥50% (or significant)** N/N total (%)	**RAS ≥70% (or severe)** N/N total (%)	**Bilateral RAS** N/N total (%)	**1-vessel coronary disease** N/N total (%)	**2-vessel coronary disease** N/N total (%)	**3-vessel coronary disease** N/N total (%)	**Left main disease** N/N total (%)
**Rihal et al. 2002**	57/297 (19.2)	21/297 (7.0)	11/297 (3.7)	42/297 (14.1)	64/297 (21.5)	74/297 (24.9)	-
**Weber et al. 2002**	19/177 (10.7)	12/177 (6.8)	8/177 (4.5)	-	-	-	-
**Yamashita et al. 2002**	21/289 (7.0)	-	3/289 (3.0)	30/289 (10.0)	26/289 (9.0)	55/289 (19.0)	-
**Aqel et al. 2003**	25/90 (28.0)	14/90 (16.0)	14/90 (16.0)	-	-	-	-
**Wang et al. 2003**	34/230 (14.8)	-	6/230 (2.6)	7/230 (3.0)	15/230 (6.5)	14/230 (6.1)	-
**Liu et al. 2004**	26/141 (18.4)	-	5/141 (3.5)	21/141 (14.9)	10/141 (7.1)	21/141 (14.9)	-
**Park et al. 2004**	109/1459 (7.5)	73/1459 (5.0)	24/1459 (1.6)	385/1459 (26.4)	271/1459 (18.6)	322/1459 (22.1)	128/1459 (8.8)
**Cohen et al. 2005**	154/843 (18.3)	99/843 (11.7)	15/843 (1.8)	125/843 (14.8)	159/843 (18.9)	201/843 (23.8)	22/843 (2.6)
**Dzielinska et al. 2006**	40/333 (12.0)	25/333 (7.5)	8/333 (2.4)	90/333 (27.0)	76/333 (22.8)	94/333 (28.2)	-
**Tumelero et al. 2006**	228/1656 (13.8)	58/1656 (3.5)	25/1656 (1.5)	-	-	-	-
**Ollivier et al. 2008**	94/650 (14.4)	-	20/650 (3.1)	219/650 (33.7)	173/650 (26.6)	152/650 (23.4)	-
**El-Mawardy et al. 2008**	19/525 (3.6)	-	-	118/525 (22.5)	78/525 (14.9)	68/525 (13.0)	6/525 (1.1)
**Przewlocki et al. 2008**	38/1036 (3.7)	26/1036 (2.5)	124/1036 (12.0)	291/1036 (28.1)	169/1036 (16.3)	173/1036 (16.7)	-
**Sani et al. 2008**	37/260 (14.2)	30/260 (11.5)	14/260 (5.4)	-	-	-	5/260 (1.9)
**Ghaffari et al. 2009**	87/732 (11.9)	35/732 (4.8)	37/732 (5.1)	100/732 (13.7)	114/732 (15.6)	220/732 (30.1)	-
**Omeish et al. 2009**	21/870 (2.4)	10/870 (1.2)	5/870 (0.6)	206/870 (23.7)	145/870 (16.7)	45/870 (5.2)	10/870 (0.2)
**Kobo et al. 2010**	41/450 (9.1)	-	-	-	-	-	-
**Rimoldi et al. 2010**	112/1403 (8.0)	-	-	-	-	-	-
**Bageacu et al. 2011**	35/450 (7.8)	15/450 (3.3)	5/450 (1.1)	-	-	-	-

**Marcantoni et al. 2011**	70/1298 (5.4)	7/1298 (0.5)	11/1298 (0.9)	278/1298 (21.4)	241/1298 (18.6)	158/1298 (12.2)	69/1298 (5.3)
**Salehi et al. 2011**	70/500 (14.0)	45/500 (9.0)	24/500 (4.8)	77/500 (15.4)	102/500 (20.4)	167/500 (33.4)	26/500 (5.2)
**Vahedparast et al. 2011**	136/481 (28.3)	-	-	88/481 (18.3)	94/481 (19.5)	170/481 (35.3)	20/481 (4.2)
**Rokni et al. 2012**	345/866 (39.8)	-	77/866 (8.9)	153/866 (17.7)	165/866 (19.1)	367/866 (42.4)	38/866 (4.4)
**Yorgun et al. 2012**	136/832 (16.4)	62/832 (7.5)	-	215/832 (25.8)	122/832 (14.6)	85/832 (10.2)	8/832 (0.96)
**Zandparsa et al. 2012**	64/165 (38.8)	-	-	50/165 (30.3)	33/165 (20.0)	82/165 (49.7)	7/165 (4.2)
**Shukla et al. 2013**	139/3500 (3.9)	-	30/3500 (0.8)	-	-	-	-
**Buller et al. 2014**	120/837 (14.3)	61/837 (7.3)	12/837 (1.4)	74/837 (8.8)	133/837 (15.9)	386/837 (46.1)	89/837 (10.6)
**Imori et al. 2014**	128/1734 (7.4)	-	-	506/1734 (29.2)	386/1734 (22.3)	206/1734 (11.9)	155/1734 (8.9)
**Khatami et al. 2014**	25/146 (17.0)	-	9/146 (6.2)	26/146 (18.0)	44/146 (30.0)	57/146 (39.0)	-
**Payami et al. 2016**	50/274 (18.2)	-	9/274 (3.3)	38/274 (18.3)	56/274 (20.4)	123/274 (44.9)	-
**Mirbolouk et al. 2019**	23/233 (9.9)	53/233 (22.7)	38/233 (16.3)	44/233 (18.9)	58/233 (24.9)	131/233 (56.2)	-

Supplemental Table S3. Detailed characteristics of each individual study included in this systematic review and meta analysis

Rihal et al. 2002

**Variables**	**Non-RAS** N=240	**RAS** N=57	**p-value**
Age (N ± SD)	64 ± 10	67 ± 11	0.09
Female sex	-	-	-
Diabetes mellitus	-	-	-
Arterial hypertension	-	-	-
Hypercholesterolemia or dyslipidemia	-	-	-
Smoking	-	-	-
Renal failure or insufficiency	-	-	-
Peripheral vascular disease (PAD)	26 (11%)	11 (19%)	0.13
1-vessel disease	-	-	-
2-vessel disease	-	-	-
3-vessel disease	-	-	-
Left main disease	-	-	-
Previous MI	-	-	-
Previous cerebrovascular accident (CVI or TIA)	24 (10%)	15 (26%)	0.001

Weber et al. 2002

Variables	Non-RAS<br>N=158	RAS<br>N=19	p-value
Age	61±11	67±8	0.004
Female sex	49 (31%)	1 (5%)	-
Diabetes mellitus	47 (30%)	13 (69%)	0.004
Arterial hypertension	103 (65%)	18 (94%)	0.009
Hypercholesterolemia or dyslipidemia	96 (61%)	15 (81%)	-
Smoking	28 (18%)	5 (25%)	-
Renal failure or insufficiency	7 (4.4%)	1 (6.2%)	-
Peripheral vascular disease	-	-	-
1-vessel disease	-	-	-
2-vessel disease	-	-	-
3-vessel disease	-	-	-
Left main disease	-	-	-
Previous MI	38 (24%)	8 (44%)	-
Previous cerebrovascular accident (CVI or TIA)	4 (2.5%)	1 (6.3%)	-

Yamashita et al. 2002

**Variables**	**Non-RAS**<br>N=268	**RAS**<br>N=21	**p-value**
Age	65.6±10.8	68.9±8.7	0.1814
Female sex	114 (43%)	10 (48%)	0.6749
Diabetes mellitus	45 (28%)	5 (24%)	0.6478
Arterial hypertension	121 (45%)	18 (86%)	0.0003
Hypercholesterolemia or dyslipidemia	83 (31%)	8 (38%)	0.5059
Smoking	83 (31%)	8 (38%)	0.4821
Renal failure or insufficiency	-	-	-
Peripheral vascular disease	-	-	-
1-vessel disease	-	-	-
2-vessel disease	-	-	-
3-vessel disease	-	-	-
Left main disease	-	-	-
Previous MI	-	-	-
Previous cerebrovascular accident (CVI or TIA)	-	-	-

Aqel et al. 2003

**Variables**	**Non-RAS**<br>N=65	**RAS**<br>N=25	**p-value**
Age	60.7±8.7	63.7±9.1	0.005
Female sex	2 (2%)	0 (0%)	-
Diabetes mellitus	35 (54%)	7 (28%)	0.035
Arterial hypertension	62 (95%)	23 (92%)	-
Hypercholesterolemia or dyslipidemia	49 (75%)	19 (76%)	-
Smoking	22 (34%)	12 (48%)	-
Renal failure or insufficiency	-	-	-
Peripheral vascular disease	18 (28%)	14 (56%)	0.015
1-vessel disease	6 (9%)	2 (8%)	-
2-vessel disease	14 (22%)	4 (16%)	-
3-vessel disease	37 (57%)	17 (68%)	-
Left main disease	-	-	-
Previous MI	7 (11%)	8 (32%)	0.015
Previous cerebrovascular accident (CVI or TIA)	-	-	-

Wang et al. 2003

Variables	Non-RAS<br>N=196	RAS<br>N=34	p-value
Age	64.2±7.8	70.6±6.3	0.001
Female sex	64 (33%)	18 (53%)	0.023
Diabetes mellitus	65 (33%)	16 (47%)	0.12
Arterial hypertension	124 (63%)	29 (85%)	0.01
Hypercholesterolemia or dyslipidemia	97 (50%)	17 (50%)	0.84
Smoking	102 (61%)	17 (50%)	0.87
Renal failure or insufficiency	-	-	-
Peripheral vascular disease	21 (11%)	6 (18%)	0.25
1-vessel disease	71 (36%)	5 (15%)	
2-vessel disease	73 (37%)	15 (44%)	0.037
3-vessel disease	52 (27%)	14 (41%)	
Left main disease	-	-	-
Previous MI	41 (21%)	11 (32%)	0.15
Previous cerebrovascular accident (CVI or TIA)	16 (8%)	3 (9%)	0.84

Liu et al. 2004

**Variables**	**Non-RAS**<br>N=115	**RAS**<br>N=26	**p-value**
Age	54±6	63±13	0.334
Female sex	71 (62%)	11 (42%)	0.07
Diabetes mellitus	15 (13%)	6 (23%)	0.321
Arterial hypertension	47 (41%)	22 (85%)	<0.01
Hypercholesterolemia or dyslipidemia	38 (33%)	15 (58%)	<0.05
Smoking	-	-	-
Renal failure or insufficiency	6 (5%)	8 (31%)	<0.01
Peripheral vascular disease	-	-	-
1-vessel disease	0	3 (12%)	-
2-vessel disease	1 (0.9%)	2 (8%)	-
3-vessel disease	0	11 (42%)	-
Left main disease	-	-	-
Previous MI	-	-	-
Previous cerebrovascular accident (CVI or TIA)	-	-	-

Park et al. 2004

Variables	Control<br>N=1301	RAS<br>N=158	p-value
Age	59.2±9.9	63.2±8.5	<0.001
Female sex	M:F = 2.05:1	M:F = 1.77:1	0.401
Diabetes mellitus	287/1301 (22%)	49/158 (31%)	<0.001
Arterial hypertension	575/1301 (44%)	104/158 (66%)	<0.001
Hypercholesterolemia or dyslipidemia	135/1227 (11%)	29/152 (19%)	0.004
Smoking	676/1299 (52%)	88/158 (56%)	0.385
Renal failure or insufficiency	53/785 (7%)	23/113 (20%)	<0.001
Peripheral vascular disease	103/1301 (8%)	43/158 (27%)	<0.001
1-vessel disease	354/1301 (27%)	31/158 (20%)	0.041
2-vessel disease	236/1301 (18%)	35/158 (22%)	0.221
3-vessel disease	256/1301 (20%)	66/158 (42%)	<0.001
Left main disease	110/1301 (9%)	18/158 (11%)	0.218
Previous MI	-	-	-
Previous cerebrovascular accident (CVI or TIA)	-	-	-

Cohen et al. 2005

**Variables**	**Non-RAS** N=689	**RAS** N=253	**p-value**
Age	63±8	68.5±7.5	<0.0001
Female sex	199 (29%)	86 (34%)	0.19
Diabetes mellitus	107 (16%)	40 (16%)	0.77
Arterial hypertension	428 (62%)	202 (80%)	0.0001
Hypercholesterolemia or dyslipidemia	441 (64%)	169 (67%)	0.45
Smoking	146 (21%)	57 (23%)	0.69
Renal failure or insufficiency	14 (2%)	46 (18%)	<0.0001
Peripheral vascular disease	64 (9%)	57 (23%)	<0.0001
1-vessel disease	-	-	-
2-vessel disease	-	-	-
3-vessel disease	-	-	-
Left main disease	-	-	-
Previous MI	116 (17%)	39 (15%)	0.79
Previous cerebrovascular accident (CVI or TIA)	-	-	-

Dzielinska et al. 2006

Variables	Non-RAS N=293	RAS N=40	p-value
Age (N ± SD)	56.6±9.5	59.8±9.6	<0.05
Female sex	85 (29.0%)	9 (22.5%)	
Diabetes mellitus	45 (15.5%)	8 (20.0%)	
Arterial hypertension	293 (100%)	40 (100%)	
Hypercholesterolemia or dyslipidemia	216 (73.7%)	35 (87.2%)	0.067
Smoking	188 (64.2%)	26 (65.0%)	
Renal failure or insufficiency	39 (13.3%)	13 (32.5%)	<0.001
Peripheral vascular disease (PAD)	223 (76.0%)	39 (97.0%)	<0.01
1-vessel disease	81 (27.6%)	9 (3.6%)	
2-vessel disease	64 (21.8%)	12 (30.0%)	
3-vessel disease	77(26.2%)	17 (42.5%)	
Left main disease	-	-	
Previous MI	-	-	
Previous cerebrovascular accident (CVI or TIA)	-	-	

Tumelero et al. 2006

Variables	Non-RAS<br>N=1428	RAS<br>N=228	p-value
Age	61.5±29.4	66.4±11.6	0.01
Female sex	646 (45%)	132 (58%)	<0.001
Diabetes mellitus	131 (9%)	37 (16%)	<0.01
Arterial hypertension	-	-	-
Hypercholesterolemia or dyslipidemia	-	-	-
Smoking	-	-	-
Renal failure or insufficiency	-	-	-
Peripheral vascular disease	-	-	-
1-vessel disease	-	-	-
2-vessel disease	-	-	-
3-vessel disease	-	-	-
Left main disease	-	-	-
Previous MI	-	-	-
Previous cerebrovascular accident (CVI or TIA)	-	-	-

El-Mawardy et al. 2008

Variables	Non-RAS N=506	RAS N=19	p-value
Age	53.2±9.5	53.8±11.6	>0.05
Female sex	99 (20%)	6 (32%)	>0.05
Diabetes mellitus	190 (38%)	7 (37%)	>0.05
Arterial hypertension	-	-	-
Hypercholesterolemia or dyslipidemia	-	-	-
Smoking	262 (52%)	10 (53%)	>0.05
Renal failure or insufficiency	-	-	-
Peripheral vascular disease	-	-	-
1-vessel disease	-	-	-
2-vessel disease	-	-	-
3-vessel disease	-	-	-
Left main disease	-	-	-
Previous MI	121 (24%)	6 (32%)	>0.05
Previous cerebrovascular accident (CVI or TIA)	8 (2%)	2 (11%)	<0.05

Ollivier et al. 2008

Variables	Non-RAS<br>N=556	RAS<br>N=94	p-value
Age (N ± SD)	67±10	69±10	NS
Female sex	119 (21.4%)	18 (19.1%)	NS
Diabetes mellitus	132 (23.7%)	26 (27.6%)	NS
Arterial hypertension	345 (62.0%)	78 (82.9%)	0.0001
Hypercholesterolemia or dyslipidemia	416 (74.8%)	70 (74.4%)	NS
Smoking	315 (56.6%)	48 (51.0)	NS
Renal failure or insufficiency	71 (12.7%)	30 (31.9)	0.001
Peripheral vascular disease (PAD)	103 (18.5%)	22 (23.4%)	NS
1-vessel disease	-	-	
2-vessel disease	-	-	
3-vessel disease	-	-	
Left main disease	-	-	
Previous MI	135 (24.2%)	26 (27.6%)	NS
Previous cerebrovascular accident (CVI or TIA)	10 (1.7%)	1 (1.0%)	0.001

Abbreviations: NS-non significant

Przewlocki et al. 2008

Variables	Non-RAS<br>N=240	RAS<br>N=56	p-value
Age	-	-	-
Female sex	-	-	-
Diabetes mellitus	-	-	-
Arterial hypertension	-	-	-
Hypercholesterolemia or dyslipidemia	-	-	-
Smoking	-	-	-
Renal failure or insufficiency	-	-	-
Peripheral vascular disease	-	-	-
1-vessel disease	272/291 (93%)	19/291 (7%)	-
2-vessel disease	155/169 (92%)	14/169 (8%)	-
3-vessel disease	155/173 (90%)	18/173 (10%)	-
Left main disease	-	-	-
Previous MI	-	-	-
Previous cerebrovascular accident (CVI or TIA)	-	-	-

Sani et al. 2008

Variables	Non-RAS<br>N=223	RAS<br>N=37	p-value
Age	56.3±8.9	62.1±10	0.001
Female sex	100 (45%)	25 (68%)	0.010
Diabetes mellitus	71 (32%)	17 (46%)	0.090
Arterial hypertension	143 (64%)	32 (87%)	0.007
Hypercholesterolemia or dyslipidemia	137 (61%)	28 (76%)	0.096
Smoking	36 (16%)	3 (8%)	0.200
Renal failure or insufficiency	-	-	-
Peripheral vascular disease	-	-	-
1-vessel disease	-	-	-
2-vessel disease	-	-	-
3-vessel disease	-	-	-
Left main disease	4 (2%)	1 (3%)	0.709
Previous MI	-	-	-
Previous cerebrovascular accident (CVI or TIA)	-	-	-

Ghaffari et al. 2009

Variables	Non-RAS<br>N=645	RAS<br>N=87	p-value
Age	59.5±9.8	58.8±8.7	0.815
Female sex	365 (57%)	51 (59%)	0.720
Diabetes mellitus	177 (27%)	31 (36%)	0.112
Arterial hypertension	-	-	-
Hypercholesterolemia or dyslipidemia	329 (51%)	45 (52%)	0.900
Smoking	132 (21%)	13 (15%)	0.225
Renal failure or insufficiency	-	-	-
Peripheral vascular disease	15 (2%)	6 (7%)	0.030
1-vessel disease	87 (14%)	13 (15%)	-
2-vessel disease	92 (14%)	22 (25%)	-
3-vessel disease	189 (29%)	31 (36%)	-
Left main disease	-	-	-
Previous MI	104 (16%)	10 (12%)	0.225
Previous cerebrovascular accident (CVI or TIA)	22 (3%)	2 (2%)	0.582

Omeish et al. 2009

Variables	Non-RAS<br>N=849	RAS<br>N=21	p-value
Age	61±11	68±8	0.040
Female sex	272 (32%)	12 (57%)	0.005
Diabetes mellitus	307 (36%)	11 (52%)	0.312
Arterial hypertension	215 (25%)	13 (62%)	0.004
Hypercholesterolemia or dyslipidemia	-	-	-
Smoking	277 (33%)	9 (43%)	0.170
Renal failure or insufficiency	-	-	-
Peripheral vascular disease	-	-	-
1-vessel disease	200 (24%)	5 (24%)	-
2-vessel disease	139 (16%)	6 (29%)	-
3-vessel disease	43 (5%)	2 (10%)	-
Left main disease	8 (1%)	2 (10%)	-
Previous MI	-	-	-
Previous cerebrovascular accident (CVI or TIA)	-	-	-

Kobo et al. 2010

Variables	Non-RAS<br>N=409	RAS<br>N=41	p-value
Age	63.5±10.3	70±9	-
Female sex	133 (33%)	26 (64%)	-
Diabetes mellitus	159 (39%)	18 (44%)	-
Arterial hypertension	409 (100%)	41 (100%)	-
Hypercholesterolemia or dyslipidemia	191 (47%)	27 (66%)	0.05
Smoking	-	-	-
Renal failure or insufficiency	41 (10%)	9 (22%)	0.005
Peripheral vascular disease	-	-	-
1-vessel disease	-	-	-
2-vessel disease	-	-	-
3-vessel disease	-	-	-
Left main disease	-	-	-
Previous MI	-	-	-
Previous cerebrovascular accident (CVI or TIA)	-	-	-

Rimoldi et al. 2010

Variables	Non-RAS<br>N=1291	RAS<br>N=112	p-value
Age	64.5±10	69.5±9	<0.0001
Female sex	595 (46%)	47 (42%)	-
Diabetes mellitus	225 (17%)	38 (34%)	<0.0001
Arterial hypertension	1291 (100%)	112 (100%)	-
Hypercholesterolemia or dyslipidemia	1037 (83%)	106 (96%)	<0.0001
Smoking	231 (18%)	28 (25%)	-
Renal failure or insufficiency	198 (17%)	53 (49%)	<0.0001
Peripheral vascular disease	88 (7%)	41 (37%)	<0.0001
1-vessel disease	-	-	-
2-vessel disease	-	-	-
3-vessel disease	-	-	-
Left main disease	-	-	-
Previous MI	-	-	-
Previous cerebrovascular accident (CVI or TIA)	72 (6%)	21 (19%)	<0.0001

Bageacu et al. 2011

Variables	Non-RAS N=416	RAS N=34	p-value
Age	-	-	-
Female sex	116 (28%)	11 (32%)	0.58
Diabetes mellitus	74 (18%)	8 (24%)	0.40
Arterial hypertension	176 (42%)	20 (59%)	0.06
Hypercholesterolemia or dyslipidemia	164 (39%)	11 (32%)	0.41
Smoking	170 (41%)	17 (50%)	0.30
Renal failure or insufficiency	-	-	-
Peripheral vascular disease	-	-	-
1-vessel disease	93 (22%)	7 (21%)	-
2-vessel disease	66 (16%)	8 (16%)	-
3-vessel disease	93 (22%)	12 (35%)	-
Left main disease	-	-	-
Previous MI	-	-	-
Previous cerebrovascular accident (CVI or TIA)	-	-	-

Marcantoni et al. 2011

**Variables**	**Non-RAS**<br>N=1228	**RAS**<br>N=70	**p-value**
Age	63±10	68±8	<0.001
Female sex	344 (28%)	27 (39%)	0.08
Diabetes mellitus	430 (35%)	34 (48%)	0.04
Arterial hypertension	1068 (87%)	69 (99%)	0.001
Hypercholesterolemia or dyslipidemia	909 (74%)	64 (91%)	0.001
Smoking	786 (64%)	39 (56%)	0.25
Renal failure or insufficiency	74 (6%)	14 (20%)	<0.001
Peripheral vascular disease	209 (17%)	32 (45%)	<0.001
1-vessel disease	332 (27%)	15 (22%)	0.33
2-vessel disease	282 (23%)	19 (27%)	0.38
3-vessel disease	209 (17%)	20 (28%)	0.03
Left main disease	86 (7%)	9 (13%)	0.09
Previous MI	332 (27%)	25 (35%)	0.17
Previous cerebrovascular accident (CVI or TIA)	86 (7%)	4 (6%)	1.00

Salehi et al. 2011

Variables	Non-RAS N=430	RAS N=70	p-value
Age	59.5±9.9	63.1±8.7	0.003
Female sex	201 (47%)	35 (50%)	0.613
Diabetes mellitus	191 (44%)	28 (40%)	0.490
Arterial hypertension	315 (73%)	58 (83%)	0.087
Hypercholesterolemia or dyslipidemia	380 (88%)	65 (93%)	0.266
Smoking	95 (22%)	13 (19%)	0.507
Renal failure or insufficiency	-	-	-
Peripheral vascular disease	-	-	-
1-vessel disease	70 (37%)	7 (49%)	0.213
2-vessel disease	85 (20%)	17 (24%)	0.424
3-vessel disease	127 (30%)	40 (57%)	<0.001
Left main disease	20 (5%)	6 (9%)	0.171
Previous MI	-	-	-
Previous cerebrovascular accident (CVI or TIA)	-	-	-

Vahedparast et al. 2011

**Variables**	**Non-RAS** N=345	**RAS** N=136	**p-value**
Age	57.87±10.51	62.73±10.81	<0.001
Female sex	171 (50%)	68 (50%)	0.789
Diabetes mellitus	108 (31%)	38 (78%)	0.775
Arterial hypertension	345 (100%)	136 (100%)	-
Hypercholesterolemia or dyslipidemia	154 (45%)	53 (39%)	0.833
Smoking	151 (44%)	50 (37%)	0.433
Renal failure or insufficiency	-	-	-
Peripheral vascular disease	-	-	-
1-vessel disease	-	-	-
2-vessel disease	-	-	-
3-vessel disease	-	-	-
Left main disease	-	-	-
Previous MI	-	-	-
Previous cerebrovascular accident (CVI or TIA)	-	-	-

Rokni et al. 2012

Variables	Non-RAS<br>N=521	RAS<br>N=345	p-value
Age	61.60±10.31	66.22±9.69	<0.001
Female sex	210 (40%)	162 (47%)	0.093
Diabetes mellitus	189 (36%)	139 (40%)	0.355
Arterial hypertension	393 (75%)	294 (85%)	0.002
Hypercholesterolemia or dyslipidemia	359 (69%)	276 (80%)	0.477
Smoking	120 (23%)	58 (17%)	0.082
Renal failure or insufficiency	63 (12%)	74 (22%)	<0.001
Peripheral vascular disease	29 (6%)	28 (8%)	0.119
1-vessel disease	103 (13%)	50 (15%)	-
2-vessel disease	94 (18%)	71 (21%)	-
3-vessel disease	182 (35%)	185 (54%)	-
Left main disease	18 (4%)	20 (6%)	0.161
Previous MI	177 (34%)	132 (38%)	0.431
Previous cerebrovascular accident (CVI or TIA)	3 (1%)	21 (6%)	0.002

Yorgun et al. 2012

Variables	Non-RAS N=696	RAS N=136	p-value
Age	60.4±8.8	65.5±10.3	0.035
Female sex	150 (22%)	28 (21%)	-
Diabetes mellitus	139 (20%)	25 (18%)	-
Arterial hypertension	696 (100%)	136 (100%)	-
Hypercholesterolemia or dyslipidemia	447 (64%)	87 (64%)	-
Smoking	368 (53%)	74 (54%)	0.042
Renal failure or insufficiency	-	-	-
Peripheral vascular disease	25 (4%)	7 (5%)	0.038
1-vessel disease	176 (25%)	39 (29%)	0.034
2-vessel disease	93 (13%)	29 (21%)	-
3-vessel disease	59 (9%)	26 (19%)	-
Left main disease	3 (0.4%)	5 (4%)	0.022
Previous MI	-	-	-
Previous cerebrovascular accident (CVI or TIA)	19 (3%)	5 (4%)	-

Zandparsa et al. 2012

Variables	Non-RAS N=101	RAS N=64	p-value
Age	59.5±9.5	60.4±9.6	0.9
Female sex	41 (41%)	37 (58%)	0.031
Diabetes mellitus	46 (46%)	25 (39%)	0.23
Arterial hypertension	64 (63%)	55 (86%)	0.002
Hypercholesterolemia or dyslipidemia	80 (79%)	49 (77%)	0.15
Smoking	34 (34%)	29 (45%)	0.13
Renal failure or insufficiency	-	-	-
Peripheral vascular disease	-	-	-
1-vessel disease	32 (32%)	18 (21%)	0.12
2-vessel disease	18 (18%)	15 (23%)	0.43
3-vessel disease	51 (50%)	31 (48%)	0.66
Left main disease	-	-	-
Previous MI	-	-	-
Previous cerebrovascular accident (CVI or TIA)	-	-	-

Shukla et al. 2013

Variables	Non-RAS<br>N=61	RAS<br>N=139	p-value
Age (N ± SD)	59.5±10.8	58.8±11.5	0.63
Female sex	18 (29.5%)	36 (25.9%)	0.76
Diabetes mellitus	8 (13.1%)	20 (14.4%)	0.81
Arterial hypertension	29 (47.5%)	106 (76.2%)	<0.001
Hypercholesterolemia or dyslipidemia	18 (29.5%)	35 (25.2%)	0.52
Smoking	7 (11.5%)	20 (14.4%)	0.58
Renal failure or insufficiency	9 (14.7%)	31 (22.7%)	-
Peripheral vascular disease (PAD)	5 (8.2%)	23 (16.5%)	0.18
1-vessel disease	16 (26.2%)	26 (18.7%)	0.31
2-vessel disease	11 (18.0%)	16 (11.5%)	0.30
3-vessel disease	20 (32.8%)	70 (50.3%)	0.03
Left main disease	7 (11.5%)	20 (14.4%)	0.94
Previous MI		-	
Previous cerebrovascular accident (CVI or TIA)		-	

Buller et al. 2014

Variables	Non-RAS<br>N=717	RAS<br>N=120	p-value
Age	67.2±9.6	71.2±7.6	0.0001
Female sex	164 (23%)	47 (39%)	0.001
Diabetes mellitus	225 (31%)	39 (33%)	0.591
Arterial hypertension	-	-	-
Hypercholesterolemia or dyslipidemia	-	-	-
Smoking	403 (56%)	73 (61%)	0.083
Renal failure or insufficiency	-	-	-
Peripheral vascular disease	-	-	-
1-vessel disease	62 (9%)	12 (10%)	-
2-vessel disease	108 (15%)	25 (21%)	-
3-vessel disease	333 (46%)	53 (44%)	-
Left main disease	108 (15%)	18 (15%)	-
Previous MI	-	-	-
Previous cerebrovascular accident (CVI or TIA)	-	-	-

Imori et al. 2014

Variables	Non-RAS<br>N=1606	RAS<br>N=116	p-value
Age	-	-	0.01
Female sex	-	-	-
Diabetes mellitus	-	-	0.38
Arterial hypertension	-	-	0.16
Hypercholesterolemia or dyslipidemia	-	-	0.17
Smoking	-	-	-
Renal failure or insufficiency	-	-	-
Peripheral vascular disease	-	-	<0.001
1-vessel disease	488 (30%)	18 (16%)	0.68
2-vessel disease	346 (22%)	40 (35%)	0.003
3-vessel disease	169 (11%)	37 (32%)	<0.001
Left main disease	134 (8%)	21 (18%)	0.004
Previous MI	-	-	0.38
Previous cerebrovascular accident (CVI or TIA)	-	-	0.93

Khatami et al. 2014

Variables	Non-RAS<br>N=109	RAS<br>N=37	p-value
Age	59.8±9.36	65.4±8.64	0.001
Female sex	27 (16%)	24 (65%)	<0.001
Diabetes mellitus	33 (30%)	21 57%)	0.004
Arterial hypertension	68 (62%)	32 (87%)	0.006
Hypercholesterolemia or dyslipidemia	56 (57%)	25 (68%)	0.087
Smoking	36 (33%)	7 (19%)	0.104
Renal failure or insufficiency	-	-	-
Peripheral vascular disease	-	-	-
1-vessel disease	-	-	-
2-vessel disease	-	-	-
3-vessel disease	-	-	-
Left main disease	-	-	-
Previous MI	-	-	-
Previous cerebrovascular accident (CVI or TIA)	-	-	-

Payami et al. 2016

Variables	Non-RAS<br>N=224	RAS<br>N=50	p-value
Age	59.8±11.1	64±10.1	0.731
Female sex	134 (60%)	32 (64%)	0.091
Diabetes mellitus	82 (37%)	16 (32%)	0.465
Arterial hypertension	224 (100%)	50 (100%)	-
Hypercholesterolemia or dyslipidemia	106 (47%)	20 (40%)	0.375
Smoking	27 (12%)	10 (20%)	0.001
Renal failure or insufficiency	18 (8%)	5 (10%)	0.336
Peripheral vascular disease	-	-	-
1-vessel disease	-	-	-
2-vessel disease	-	-	-
3-vessel disease	-	-	-
Left main disease	-	-	-
Previous MI	-	-	-
Previous cerebrovascular accident (CVI or TIA)	-	-	-

Mirbolouk et al. 2019

**Variables**	**Non-RAS**<br>N=157	**RAS**<br>N=76	**p-value**
Age	64±10	64.5±8	0.019
Female sex	88 (56%)	42 (55%)	0.156
Diabetes mellitus	74 (47%)	36 (47%)	0.581
Arterial hypertension	121 (77%)	58 (76%)	0.986
Hypercholesterolemia or dyslipidemia	97 (62%)	53 (70%)	0.892
Smoking	-	-	-
Renal failure or insufficiency	14 (9%)	17 (22%)	<0.001
Peripheral vascular disease	-	-	-
1-vessel disease	34 (22%)	10 (13%)	-
2-vessel disease	37 (25%)	21 (28%)	-
3-vessel disease	86 (55%)	45 (59%)	-
Left main disease	-	-	-
Previous MI	28 (18%)	18 (24%)	0.339
Previous cerebrovascular accident (CVI or TIA)	-	-	-

Supplemental Table S4: Study quality assessment using the Ottawa-Newcastle Scale for observational/cohort studies

** A maximum points that an individual study can score is 9, each category can maximally be assigned with 1 star while "comparability" domain can be graded with maximum of 2 stars*

Study	Representativness of cohort	Selection of non-exposed cohort	Ascertainment of exposure	Demostration that outcomes were not present at start of study	Comparability of the cohort	Outcome ascertainment	Adequacy of length of follow up	Extent of missing data	Total stars*
Rihal et al. 2002	*	*	*	-	**	*	*	*	**8**
Weber et al. 2002	*	*	-	-	*	*	*	*	**6**
Yamashita et al. 2002	*	*	*	-	**	*	*	*	**8**
Aqel et al. 2003	*	*	*	-	**	*	*	*	**8**
Wang et al. 2003	*	*	*	-	*	*	*	*	**7**
Liu et al. 2004	*	*	*	-	*	*	*	*	**7**
Park et al. 2004	*	*	*	-	*	*	*	*	**7**
Cohen et al. 2005	*	*	*	-	**	*	*	*	**8**
Dzielinska et al 2006	*	*	*	-	**	*	*	*	**8**
Tumelero et al. 2006	*	*	*	-	*	*	*	*	**7**
Ollivier et al. 2008	*	*	*	-	*	*	*	*	**7**
El-Mawardy et al. 2008	*	*	*	-	*	*	*	*	**7**

Przewlocki et al. 2008	*	*	*	-	*	*	*	*	**7**
Sani et al. 2008	*	*	*	-	*	*	*	*	**7**
Ghaffari et al. 2009	*	*	*	-	*	*	*	*	**7**
Omeish et al. 2009	*	*	*	-	*	*	*	*	**7**
Kobo et al. 2010	*	*	*	-	**	*	*	*	**8**
Rimoldi et al. 2010	*	*	*	-	**	*	*	*	**8**
Bageacu et al. 2011	*	*	*	-	*	*	*	*	**7**
Marcantoni et al. 2011	*	*	*	-	*	*	*	*	**7**
Salehi et al. 2011	*	*	*	-	*	*	*	*	**7**
Vahedparast et al. 2011	*	*	*	-	*	*	*	*	**7**
Rokni et al. 2012	*	*	*	-	*	*	*	*	**7**
Yorgun et al. 2012	*	*	*	-	*	*	*	*	**7**
Zandparsa et al. 2012	*	*	*	-	*	*	*	*	**7**
Shukla et al. 2013	*	*	*	-	*	*	*	*	**7**
Buller et al. 2014	*	*	*	-	**	*	*	*	**8**
Imori et al. 2014	*	*	*	-	**	*	*	*	**8**
Khatami et al. 2014	*	*	*	-	*	*	*	*	**7**

Payami et al. 2016	*	*	*	-	*	*	*	*	**7**
Mirbolouk et al. 2019	*	*	*	-	**	*	*	*	**8**
